# Comparison of In Situ and Postsynthetic Formation of MOF-Carbon Composites as Electrocatalysts for the Alkaline Oxygen Evolution Reaction (OER)

**DOI:** 10.3390/molecules30020208

**Published:** 2025-01-07

**Authors:** Linda Sondermann, Laura Maria Voggenauer, Annette Vollrath, Till Strothmann, Christoph Janiak

**Affiliations:** Institut für Anorganische Chemie und Strukturchemie, Heinrich-Heine-Universität Düsseldorf, 40225 Düsseldorf, Germany; linda.sondermann@hhu.de (L.S.); lavog108@uni-duesseldorf.de (L.M.V.); annette.vollrath@uni-duesseldorf.de (A.V.); till.strothmann@hhu.de (T.S.)

**Keywords:** metal–organic frameworks (MOFs), electrocatalysis, oxygen evolution reaction (OER), nickel, iron, carbon nanotubes, ketjenblack, carbon paper, in situ, postsynthetic

## Abstract

Mixed-metal nickel-iron, Ni_x_Fe materials draw attention as affordable earth-abundant electrocatalysts for the oxygen evolution reaction (OER). Here, nickel and mixed-metal nickel-iron metal–organic framework (MOF) composites with the carbon materials ketjenblack (KB) or carbon nanotubes (CNT) were synthesized in situ in a one-pot solvothermal reaction. As a direct comparison to these in situ synthesized composites, the neat MOFs were postsynthetically mixed by grinding with KB or CNT, to generate physical mixture composites. The in situ and postsynthetic MOF/carbon samples were comparatively tested as (pre-)catalysts for the OER, and most of them outperformed the RuO_2_ benchmark. Depending on the carbon material and metal ratio, the in situ or postsynthetic composites performed better, showing that the method to generate the composite can influence the OER activity. The best material Ni_5_Fe-CNT was synthesized in situ and achieved an overpotential (*η*) of 301 mV (RuO_2_ *η* = 354 mV), a Tafel slope (*b*) of 58 mV/dec (RuO_2_ *b* = 91 mV/dec), a charge transfer resistance (R_ct_) of 7 Ω (RuO_2_ R_ct_ = 39 Ω), and a faradaic efficiency (FE) of 95% (RuO_2_ FE = 91%). Structural changes in the materials could be seen through a stability test in the alkaline electrolyte, and chronopotentiometry over 12 h showed that the derived electrocatalysts and RuO_2_ have good stability.

## 1. Introduction

Nickel-based materials have emerged as promising electrocatalysts for the oxygen evolution reaction (OER), one of the two half-reactions in electrochemical water splitting (OER, alkaline conditions: 4OH^−^ → O_2_ + 4e^−^ + 2H_2_O, E° = 1.23 V vs. the reversible hydrogen electrode, RHE) [1,2,3]. The OER is the bottleneck of electrochemical water splitting and requires a higher activation energy than the hydrogen evolution reaction (HER, alkaline conditions: 4H_2_O + 4e^−^ → 2H_2_ + 4OH^−^, E° = 0.00 V vs. RHE) [2]. To produce one oxygen molecule in the OER, four electron-proton coupled reaction steps, which require a larger applied potential than the theoretical potential of 1.23 V for electrochemical water splitting, have to be gone through [3,4]. The difference between the applied potential (E) and the theoretical potential of E° = 1.23 V, which still needs to be corrected for the internal resistance (IR) because of the ohmic drop, is called overpotential *η* (*η* = E − E° (1.23 V) − IR) [5]. The overpotential is a parameter used to check the efficiency of an electrocatalyst and is determined at a defined current density, with often 10 mA/cm^2^ being used as a reference point [2,6].

Currently, noble-metal-based catalysts like ruthenium oxide (RuO_2_) and iridium oxide (IrO_2_) are seen as benchmark electrocatalysts for the OER [7,8]. RuO_2_ is known to efficiently drive the OER in alkaline and acidic media [9,10,11]. However, the high costs due to the scarcity of the noble metals impede large-scale industrial utilization of these electrocatalysts [12]. Nickel presents an attractive option for the OER due to its abundance, lower cost, and electrocatalytic activity [3,12,13]. Ni^3+^ is assumed to be the main active site in nickel-based materials, leading to improved conductivity and accelerated reaction kinetics [8,12,14,15]. The incorporation of iron into nickel-based materials can be advantageous for electrocatalytic water splitting because iron promotes the nickel oxidation from Ni^2+^ to Ni^3+^ [15,16,17]. In recent years, various nickel(-iron)-based materials, including oxides, (oxy)hydroxides, phosphides, sulfides, nitrides and metal–organic frameworks (MOFs), have been extensively studied for their electrocatalytic ability in the OER [1,3,18]. MOFs usually act as pre-catalysts that only form the active phase under the electrochemical reaction conditions [19]. The resulting electrocatalysts, often metal (oxy)hydroxides, can potentially retain some of the porosity of the MOFs, which helps in lessening diffusion limitations and increasing the electrocatalytic activity of the material [12,19,20,21]. For mixed-metal MOFs, the intimate and uniform mixture of the involved metals is retained at the nanoscale in the derived electrocatalyst.

MOFs and also the derived metal (oxy)hydroxide electrocatalysts have low electrical conductivity, which needs to be increased through the combination with a carbon material [22,23,24]. The admixture of a carbon material, that is, the formation of a MOF/carbon composite, improves the efficiency of charge transfer processes in electrochemical applications [25]. Ketjenblack (KB) or carbon nanotubes (CNTs) are often utilized to produce composite materials. KB is a carbon black material and displays good electrical conductivity, a high surface area and thermal stability [22,26,27,28]. CNTs are cylindrical nanostructures composed of carbon atoms arranged in a hexagonal lattice, forming seamless tubes with diameters typically on the order of nanometers. CNTs have good mechanical stability, high thermal stability, and high electrical and thermal conductivities, which make them interesting to use as an alternative to carbon black materials in composites [25,29,30,31]. Preceding research presented different highly active MOF-composite-derived materials for the alkaline OER [32,33,34,35].

The MOF and the carbon material can be combined in situ, that is, by carrying out the MOF synthesis in the presence of the carbon material, or postsynthetically, that is, by combining the separately prepared MOF with the carbon material through physical mixing [36]. Both routes are used in the literature but mostly without direct comparison. Studies on in situ prepared Ni-MOF/carbon composites [37] are, of course, difficult to compare with studies on postsynthetically prepared physical mixtures [38]. Few Ni-MOF/carbon works on OER include a comparison of both routes [39].

Here, we present the direct comparison of Ni(Fe)-MOF/carbon composites that were prepared in situ and postsynthetically with the same weight percent of carbon with respect to their electrocatalytic activity as electrode material in the oxygen evolution reaction. The parent MOF was Ni-BTC, with the formula [Ni_3_(BTC)_2_(Me_2_NH)_3_]·(DMF)_4_(H_2_O)_4_ (BTC = benzene-1,3,5-tricarboxylate, DMF = N,N-dimethylformamide) [40]. The parent MOF Ni-BTC has the same structure as HKUST-1 (HKUST-1 = Hong Kong University of Science and Technology-1), also known as Cu-BTC or MOF-199 [41,42]. In both MOFs, dinuclear metal(II) {M_2_(OOC-)_4_} paddle-wheel secondary building units (SBUs) with the M_2_ units coordinated by four carboxylate groups from four BTC linkers are assembled into a three-dimensional network, as depicted in Supplementary Figure S1 [40,41,42].

## 2. Results and Discussion

### 2.1. Synthesis and Characterization of the MOF Composites

**Figure 1:**
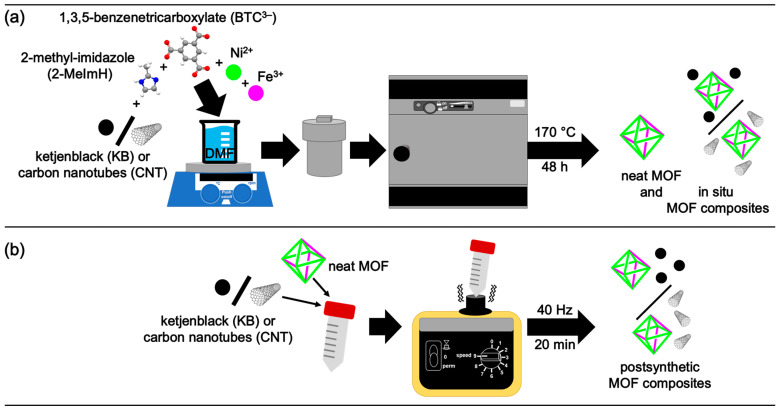
Schematic illustration of the (**a**) in situ MOF composite synthesis and (**b**) postsynthetic MOF composite formation. The building unit and packing diagram of the parent Ni-MOF (Ni-BTC) are shown in Supplementary Figure S1. In the structures of BTC^3−^ and 2-MeImH the carbon atoms are depicted gray, nitrogen blue, hydrogen white and oxygen red.

For the in situ synthesis, the nickel and nickel-iron MOF composites were synthesized by a simple one-pot solvothermal reaction at 170 °C for 48 h from Ni(NO_3_)_2_·6H_2_O or a mixture of Ni(NO_3_)_2_·6H_2_O, Fe(NO_3_)_3_·9H_2_O (molar Ni:Fe ratio 10:1 or 5:1), 1,3,5-benzenetricarboxylic acid (H_3_BTC), 2-methylimidazole (2-MeImH), and ketjenblack (KB) or carbon nanotubes (CNT) in *N,N*-dimethylformamide (DMF) (Figure 1a). The amount of carbon materials was set to give about 10% of KB or CNT in the composite.

For the postsynthetic admixture, the neat Ni-, Ni_10_Fe, and Ni_5_Fe-MOFs were generated through the same procedure as noted above [40], but without the presence of a carbon material. The neat MOFs were physically mixed with 10 wt% of KB or CNT with respect to the MOF amount using a vortex mixer at 40 Hz for 20 min (Figure 1b).

A hyphen is used for the in situ MOF composites, i.e., Ni_X_Fe-CNT or Ni_X_Fe-KB depending on the used carbon material. A slash indicates the postsynthetically physically mixed MOFs/carbon materials, i.e., Ni_X_Fe/CNT or Ni_X_Fe/KB.

To assess the influence of iron on the electrocatalytic performance, the Ni-only MOF and its carbon composites were prepared and investigated in comparison to the Ni_X_Fe materials.

The verification of the MOF synthesis had to be carried out for the neat MOFs and for the in situ prepared MOF-carbon composites. For the latter, it had to be determined if and to what extent the presence of carbon would influence the MOF crystallinity and porosity. The crystallinity was assessed by powder X-ray diffraction (PXRD), and the surface area determination and porosity were calculated by BET and NLDFT on the volumetric gas sorption isotherms. For the postsynthetically admixed MOF/carbon composites, PXRD and gas sorption measurements confirmed the superposition of the MOF and carbon contributions.

**Figure 2:**
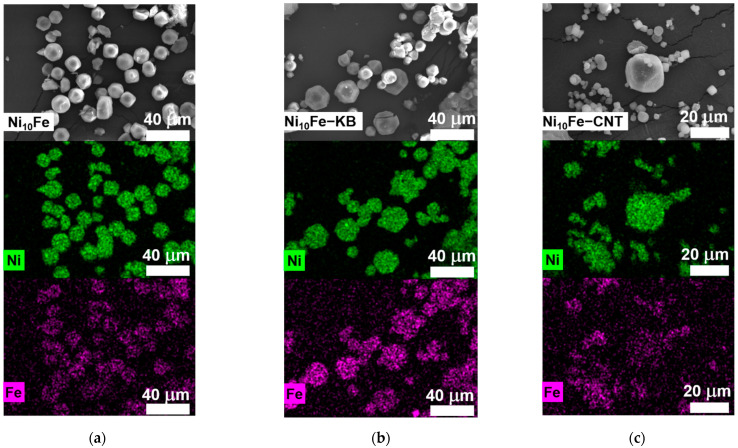
SEM images (first row) and EDX elemental mapping for Ni (second row) and Fe (third row) for (**a**) Ni_10_Fe, (**b**) Ni_10_Fe-KB, and (**c**) Ni_10_Fe-CNT. Further SEM images and the SEM-EDX spectra are displayed in Supplementary Figures S2–S9. Note the smaller scale for the CNT composite vs. the neat MOF and MOF-KB composite.

It was verified by energy-dispersive X-ray spectroscopy in the scanning electron microscope (SEM-EDX) of the reaction products that the used molar 10:1 and 5:1 nickel-to-iron ratios in the in situ synthetic procedure were also present in the nickel-iron containing MOFs and their composites (Supplementary Table S1). SEM-EDX element mapping displays a homogeneous distribution of Ni and Fe inside the samples (Figure 2 and Supplementary Figures S2–S4). The morphology of the MOF particles ranges from an octahedral to a cuboctahedral shape. The change in shape was also seen by Qiao et al. and correlated with the iron content in their samples [43]. Noteworthy, the average particle size for the in situ MOF-CNT composites (Ø(Ni-CNT) = 16 µm, Ø(Ni_10_Fe-CNT) = 6 µm, Ø(Ni_5_Fe-CNT) = 6 µm) is smaller than for the neat MOF (Ø(Ni) = 27 µm, Ø(Ni_10_Fe) = 15 µm, Ø(Ni_5_Fe) = 7 µm) and the in situ MOF-KB composites (Ø(Ni-KB) = 27 µm, Ø(Ni_10_Fe-KB) = 14 µm, Ø(Ni_5_Fe-KB) = 15 µm) (Figure 2 and Figures S2–S9).

Powder X-ray diffraction (PXRD) patterns of the neat MOFs, the in situ, and postsynthetically formed composites present the same reflexes as the simulated pattern of the structurally authenticated Ni-MOF (Ni-BTC) in Figures S1, S10 and S11 (see Supplementary Figure S1 for the building unit and packing diagram of the parent MOF Ni-BTC). Thereby, the in situ incorporation of KB had less influence on the MOF crystallinity than did CNT. The width of the reflections and signal-to-noise ratio of the KB samples was similar to those in the neat MOF diffractograms. For the in situ CNT samples, the reflexes were broader and the noise increased. The lower crystallinity of the in situ CNT samples correlates with the seemingly smaller crystallites which formed in the presence of CNT (Figure 2, Supplementary Figures S2–S9). In addition, there could be more defects in the MOF-CNT composite samples [44,45,46,47]. Mazlan et al. reviewed recent works on the growth of MOFs in the presence of graphene oxide and also showed that depending on the MOF, the functional groups of the graphene oxide (GO), and the synthesis method of the composite, the crystal growth of the MOFs could be influenced to result in ordered or disordered (nanosized) MOF-GO structures [47]. No additional reflexes other than those for Ni-BTC were detected in the Ni_x_Fe-MOF samples, which indicates that iron was well incorporated into the structure of the Ni-MOF. KB presented three broad diffraction peaks at 8, 26 and 44° matching the (100), (002), and (101) planes of graphitized carbon, respectively [22,48]. CNT displayed its two broad diffraction peaks at 26° (002) and 44° (101) [49].

Thermogravimetric analysis (TGA) of the neat MOFs and their in situ composites was performed on the as-synthesized samples without activation and under N_2_ atmosphere (Supplementary Figure S12a–c). Two major mass loss steps were recorded, where the first one at 350 °C can be attributed to the evaporation of coordinated and crystal solvent molecules from the porous Ni-BTC structure (~20–30% mass loss) [40,50,51,52,53]. The second mass loss step after 350 °C can be ascribed to the decomposition of the BTC-linker and the disintegration of the MOF structure (~45–55% mass loss) [40,50,51,52,53]. The carbon materials KB and CNT show good stability until 600 °C under the measurement conditions (Supplementary Figure S12d). In general, the residual masses of the MOFs without any carbon material are lower than their corresponding composites, which can be used to calculate the amount of carbon material inside the in situ composites via subtraction of the residual mass of the neat MOFs from the residual mass of their composites (Supplementary Table S2).

Fourier transform infrared (FT-IR) spectra (Supplementary Figure S13) of the in situ composite materials display the characteristic bands of the neat Ni-BTC MOF (Supplementary Table S3). All spectra show a broad band in the region of 3600–3000 cm^–1^, which can be ascribed to the stretching vibration of the hydroxy group from adsorbed or coordinated water (Supplementary Figure S1) [40,54,55]. The samples were not activated before the FT-IR measurement. The bands around 1629–1559 cm^–1^ and 1440–1345 cm^–1^ can be assigned to the asymmetric and symmetric stretching vibrations of carboxylate groups [24,51,54,56]. Characteristic metal–oxygen bond vibrations can be seen for a mixed-metal–oxygen (FeNi-O, Fe_2_Ni-O) bond around 722–713 cm^–1^ (Lit. ~720 cm^–1^ [57,58]) and a Fe/Ni–O bond around 589–453 cm^–1^ (Lit. 587–461 cm^–1^ [24,35,57,59]) in all spectra.

To examine the porosity of the materials, N_2_-sorption measurements were conducted. The N_2_-adsorption isotherms at 77 K were used to calculate the specific Brunauer–Emmett–Teller (BET) surface areas (S(BET)) and pore volumes of the materials (Figures S14–S17), and they are summarized in Supplementary Tables S4 and S5.

The isotherm shape of the neat Ni-MOF and Ni_10_Fe-MOFs is close to type I, with a slight type II increase with pressure and a slight H4 hysteresis. The distinct uptake at low relative pressure originates from the filling of the micropores. The type II branch and the hysteresis loops derive here from the texture effect of the physisorption in the meso- and macroporous voids of the aggregated crystallites [60,61]. Ni_5_Fe demonstrates a steeper uptake approaching a relative pressure of 1 with a more pronounced H3 hysteresis loop than the other neat MOF samples, which could be due to a stronger capillary condensation in this sample and is an indication that the sample exhibits more aggregates and larger pores from defects, which are not completely filled with pore condensate [61].

The isotherms of the neat carbon materials are displayed in Figure S14d. KB is a mesoporous carbon material (S(BET) = 1415 m^2^/g, pore volume: 1.59 cm^3^/g, pore size: ~4 ± 2 nm, Supplementary Figure S15d). KB shows a mixed adsorption isotherm consisting of an IUPAC type I and II branch at low and high relative pressure, respectively, with an H3 hysteresis at p/p_0_ > 0.9 and H4 hysteresis between 0.4 < p/p_0_ < 0.9 [61]. CNT is a carbon material, where the inner surface area of the tubes is not accessible and only their outer surface area was measured (S(BET) = 117 m^2^/g, Supplementary Figure S6d) [62]. CNT gives a type III isotherm with a slight H3 hysteresis, characteristic of nonporous or macroporous materials [61].

The MOF-KB isotherms are evidently a superposition of the neat MOF and KB isotherms and have the same KB-characteristic type II branch at high relative pressure with the H3 and H4 hysteresis upon desorption. There are also bimodal pore size distributions with maxima at ~2 nm and ~4 nm (Supplementary Figures S15 and S17). The BET surface areas for the MOF samples and their in situ composites fit into the range of published values for Ni-BTC and their analogs (Lit. 86–596 m^2^/g [35,59,63], Ni-MOF: 648 m^2^/g, Ni-KB: 414 m^2^/g, Ni-CNT: 301 m^2^/g, Ni_10_Fe: 859 m^2^/g, Ni_10_Fe-KB: 526 m^2^/g, Ni_10_Fe-CNT: 387 m^2^/g, Ni_5_Fe: 698 m^2^/g, Ni_5_Fe-KB: 599 m^2^/g, Ni_5_Fe-CNT: 67 m^2^/g) [28]. The sorption isotherms of the postsynthetic composites show a similar curvature as the in situ synthesized composites (Supplementary Figure S16, Table S5).

### 2.2. Electrocatalytic Results

**Figure 3:**
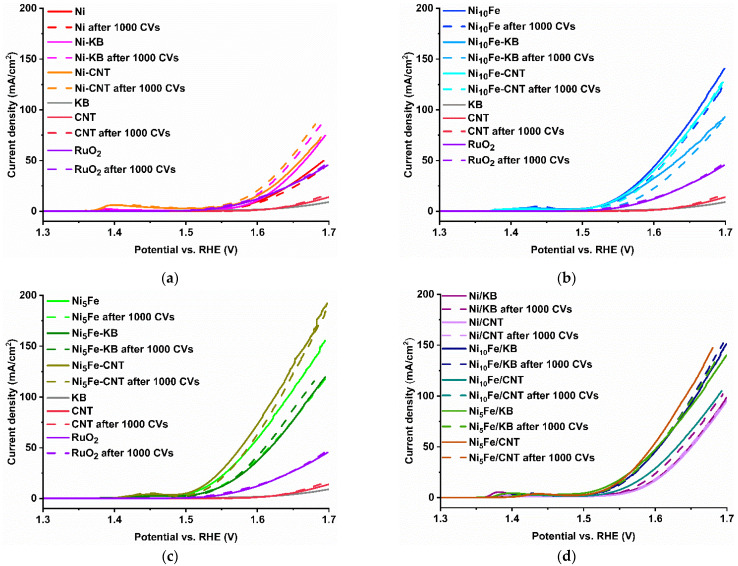
LSV plots before and after 1000 CVs of (**a**) Ni-MOF (Ni), (**b**) Ni_10_Fe, (**c**) Ni_5_Fe with their in situ carbon composites compared to KB, CNT, and RuO_2_, (**d**) postsynthetic physical mixtures of Ni- and Ni_x_Fe-MOFs with KB or CNT.

**Figure 4:**
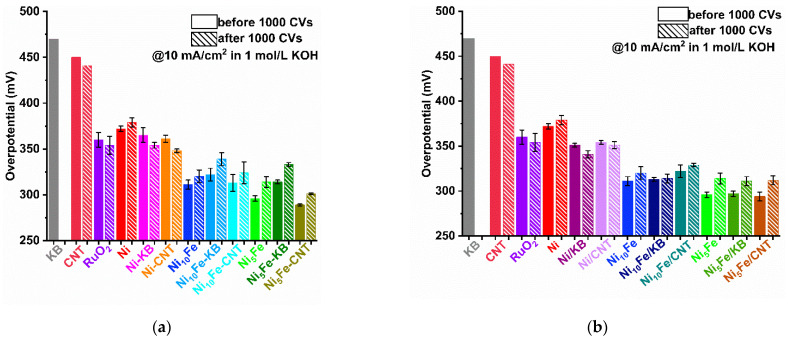
Overpotentials *η*_10_ calculated from LSV curves in Figure 3a–d of (**a**) MOFs and in situ MOF-carbon samples, (**b**) MOFs and postsynthetic MOF/carbon mixtures; each with KB, CNT, and RuO_2_ for comparison. No *η*_10_ value of KB could be determined after 1000 CVs because KB did not reach 10 mA/cm^2^ anymore. Error bars are derived from the highest and lowest achieved *η*_10_ value in multiple measurements.

**Figure 5:**
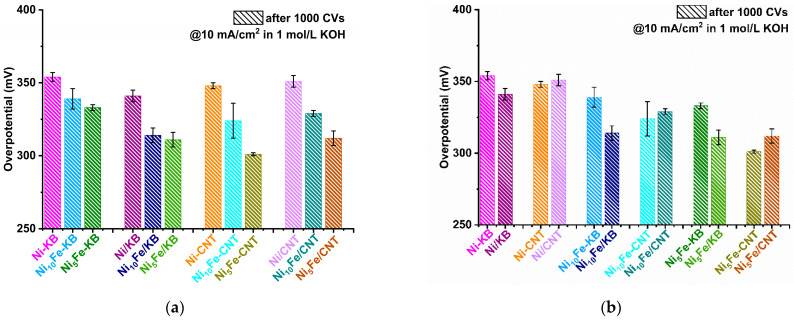
Overpotentials *η*_10_ calculated from LSV curves in Figure 3 of the in situ MOF-carbon samples and the postsynthetic MOF/carbon mixtures for comparison: (**a**) grouped according to the same three KB and CNT composites each, (**b**) grouped according to Ni-MOF, Ni_10_Fe, and Ni_5_Fe and their composites. For clarity, only the data after 1000 CVs are presented (see Supplementary Figure S26 for the additional data before the 1000 CVs and Figure S24 for LSV plots grouped according to the carbon composites). Error bars are derived from the highest and lowest achieved *η*_10_ value in the multiple measurements.

**Figure 6:**
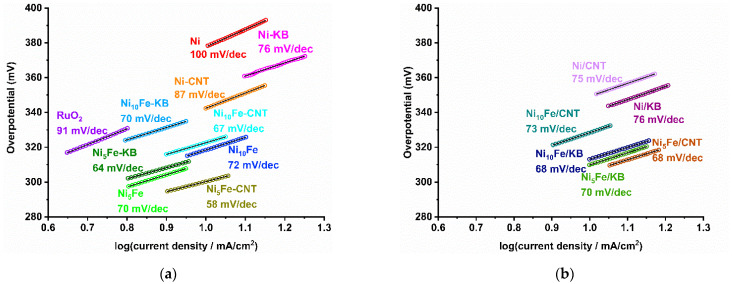
Tafel plots with Tafel slope *b* (given in mV/dec) of (**a**) MOFs, in situ MOF-carbon samples, and RuO_2_ and (**b**) postsynthetically mixed MOF/carbon composites.

**Figure 7:**
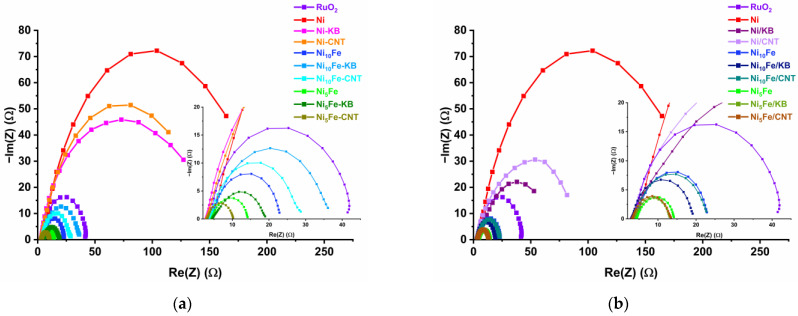
Nyquist plots of RuO_2_, pristine MOFs, and (**a**) in situ and (**b**) postsynthetically mixed composites. The inset is a magnification between Re(Z) = 0–40 Ω.

Multiple electrochemical measurements were performed to assess the electrocatalytic ability of the neat MOF, in situ, and postsynthetically mixed carbon composite samples in the alkaline (1 mol/L KOH electrolyte, pH = 14) towards the oxygen evolution reaction (OER). A three-electrode setup with an ink-coated carbon paper as the working electrode was utilized (see experimental section for details). MOFs are usually of low stability in alkaline medium, where they form metal hydroxides. Metal hydroxides are recognized as electrocatalytically active species. Mixed-metal MOFs allow achieving a defined mixed-metal hydroxide composition with a uniform distribution of the metals on the nano level [64,65,66,67]. From activated linear sweep voltammetry (LSV) measurements (Figure 3) the electrochemical parameters overpotential (*η*) (Figure 4 and Figure 5 and Supplementary Figure S26) and Tafel slope (*b*) (Figure 6 and Supplementary Figure S27a) were derived. A comparison of the overpotentials before and after 1000 cyclic voltammetry cycles (CVs) and a 12 h chronopotentiometry (Supplementary Figure S31) was carried out to test the stability of the electrocatalysts. The electrochemical parameter charge transfer resistance (R_ct_) was achieved through electrochemical impedance spectroscopy (EIS) (Figure 7 and Supplementary Figure S27b). Measurement details are given in the experimental section.

The electrocatalytic performance of the electrocatalysts derived from the neat MOFs, the in situ synthesized MOF-carbon composites, and the postsynthetic mixtures of Ni-MOF, Ni_10_Fe, and Ni_5_Fe were contrasted with the performance of the RuO_2_ benchmark.

The LSV plots in Figure 3 demonstrate that Ni_5_Fe-CNT achieves the highest current density (*j*) of all samples before and after the stability test. All samples except for Ni-MOF reached higher current densities than the commercial RuO_2_ and the neat carbon materials KB and CNT. Regarding the current density, a higher iron content in the composites allows attaining higher current densities at a given potential. Regarding the type of carbon material, in the case of the postsynthetically mixed Ni_10_Fe composites, KB managed to gain a slightly higher current density than CNT, but in the case of the Ni_5_Fe-BTC composites, the CNT composite reaches a higher current density (Figure S25). For most samples, the current densities before and after the stability test were similar or only slightly lower after 1000 CV cycles, except for the Ni-MOF composites, where higher current densities were attained after the 1000 CV cycles. The oxidation of Ni^2+^ to Ni^3+^ is visible by the peak around and above 1.4 V vs. RHE (Figure 3), and shifts to higher potentials when iron is included in the samples, which indicates a stabilization of the Ni^2+^ state [57,68].

From the potential at the current density of 10 mA/cm^2^ (E_10_), which is often chosen for comparison, the overpotential (*η*_10_) is derived by subtracting the standard potential of 1.23 V, i.e., *η*_10_ = E_10_ − 1.23 V. The overpotentials before and after the 1000 CV cycle stability test are summarized in the bar diagrams in Figure 4 (values are listed in Supplementary Table S6). The initial overpotential of Ni-MOF is 372 mV, and after the stability test, it has slightly increased to 379 mV. With the addition of a carbon material, the overpotential for Ni-MOF decreases, and unexpectedly is lowered further after the CV cycles, both in situ and more so when postsynthetically mixed [35,69].

In general, the addition of Fe into the Ni-MOF at a 5:1 molar Ni:Fe ratio led to a significant and anticipated [68,70] decrease of the overpotential, down to below 300 mV before the activation cycles (Ni_5_Fe: *η*_10_ *=* 296 mV, Ni_5_Fe/KB: 297 mV, Ni_5_Fe-CNT *η*_10_ *=* 289 mV, Ni_5_Fe/CNT: 294 mV) (Figure 4 and Supplementary Table S6). Activation with 1000 CV cycles then gives the normally seen increase in overpotential to values between 300 and 340 mV. Still, all Ni_x_Fe-MOF-carbon samples outperformed the benchmark RuO_2_ (*η*_10_ *=* 360 mV, before to 354 mV, after activation). The overall lowest overpotential, i.e., highest activity for OER, is given by Ni_5_Fe-CNT with *η*_10_ = 289 mV, before to 301 mV, after activation. It can be clearly seen that an increase in Fe content from the Ni_10_Fe to the Ni_5_Fe series is advantageous, with the latter giving lower overpotentials (Figure 5a).

Judged by the lower overpotential, the in situ synthesized CNT composites perform better than the in situ KB composites (Figure 4a, Supplementary Table S6). Comparing the in situ and the respective postsynthetically mixed composites, for KB, the postsynthetic ones, and for CNT, the in situ ones have the lower overpotential (Figure 5b).

The correlation of the overpotential from the LSV curves in Figure 3 with the decadic logarithm of the current density is the Tafel plot (Figure 6 and Figure S27a), in which the data points can be fitted by the linear Tafel equation (*η* = *a* + *b* × log(*j*) ) with slope *b* (tabulated in Supplementary Table S6) [71]. For the Tafel lines, we tried to choose regions around the overpotentials needed to reach 10 mA/cm^2^, while ensuring clear linearity in the Tafel coordinates. A smaller Tafel slope *b* represents a steeper increase in current density together with a smaller increase in overpotential. Both suggest a faster reaction rate and better electrocatalytic performance [72]. The Tafel plots of RuO_2_ and the MOF samples are presented in Figure 6. The Tafel plot of KB and CNT is displayed in Supplementary Figure S27a. The smallest Tafel slope is seen for Ni_5_Fe-CNT with 58 mV/dec, but for all MOFs and their carbon composites, the Tafel slopes lie in a narrow range of 64 to 76 mV/dec, except for neat Ni-MOF (100 mV/dec) and Ni-CNT (87 mV/dec). Thus, the Tafel slopes of the Ni_x_Fe-MOF carbon composites, both in situ and postsynthetically mixed, are much smaller than for RuO_2_ (*b* = 91 mV/dec [60]) or the neat carbon materials (KB *b* = 134 mV/dec, CNT *b* = 137 mV/dec). The Tafel slopes from our study agree with published values of comparable materials such as Ni(Fe)(OH)_2_/KB *b* = 66 mV/dec [28], FeNiNH_2_BDC MOF@2–6 wt% CNT *b* = 69–84 mV/dec [57], and Ni-BTC *b* = 114 mV/dec [73]. Yet, Tafel values must be seen critically with regard to the characterization of the catalyst because the values can be influenced depending on the chosen data range. Therefore, the entire current-potential data in Tafel coordinates are presented in Supplementary Figures S28–S30.

The Tafel slope also provides information on the rate-determining step (rds) according to Equations (1)–(4) (M = active site) when assuming Krasil’shchikov’s OER mechanism [71,74]. The Tafel slope of 58 mV/dec for Ni_5_Fe-CNT and the range of Tafel slopes of 64 to 76 mV/dec for the other Ni_x_Fe-MOF composites suggest that the metal hydroxide deprotonation reaction (Equation (2)) with *b* = 60 mV/dec is the rate-limiting step.


(1) $$\begin{array}{ll} \;M+{\mathrm{OH}}^{-}\;⇄\;\mathrm{MOH}+e^{-} & \;b=120\;\mathrm{mV}/\mathrm{dec} \end{array}$$

(2) $$\begin{array}{ll} \mathrm{MOH}+{\mathrm{OH}}^{-}⇄\;{\mathrm{MO}}^{-}+H_{2}O & \;b=60\;\mathrm{mV}/\mathrm{dec} \end{array}$$

(3) $$\begin{array}{ll} \;{\mathrm{MO}}^{-}\;\to \;\mathrm{MO}+e^{-} & \;b=45\;\mathrm{mV}/\mathrm{dec} \end{array}$$

(4) $$\begin{array}{ll} \;2\;\mathrm{MO}\;\to \;2\;M+O_{2} & \;b=19\;\mathrm{mV}/\mathrm{dec} \end{array}$$

We used the widely common analysis and interpretation of the Tafel slopes as one parameter of many to define the electrocatalytic activity of our materials. Tafel slopes alone are not sufficient to clearly state that one catalyst is better than the another. Other parameters like overpotential at a defined current density, charge transfer resistance, and faradaic efficiency are needed to better understand the electrocatalytic activity of a sample, and the Tafel slope values support the aforementioned more relevant OER parameters. The charge transfer resistance (R_ct_) provides insight into the OER kinetics. A low R_ct_ indicates a fast charge transfer rate during a redox reaction [75]. The R_ct_ values were obtained by electrochemical impedance spectroscopy (EIS), which was conducted at 1.5 V vs. RHE in a frequency range of 0.01 Hz to 10 kHz at an alternating current (AC) with a potential amplitude of 10 mV. The resulting Nyquist plots, fitted to a simple Randles cell model, are presented in Figure 7 and Supplementary Figure S27b [76]. The diameter of the displayed semi-circles gives insight into the R_ct_ value; a smaller semi-circle indicates a lower R_ct_ and, following, faster OER kinetics [20,77].

The mixed-metal Ni_x_Fe-MOF samples and their carbon composites exhibit much lower R_ct_ values (<33 Ω) than neat Ni-MOF and its composites (R_ct_ 80 Ω, Supplementary Table S7). The mixed-metal Ni_x_Fe-MOF samples and their carbon composites differ primarily by the Ni:Fe ratio, with the Ni_10_Fe materials between 17–33 Ω and the Ni_5_Fe materials between 7–15 Ω, such that the carbon composites vary around the value of the neat MOF (Ni_10_Fe, R_ct_ = 20 Ω and Ni_5_Fe, R_ct_ = 10 Ω). Overall, the in situ synthesized Ni_5_Fe-CNT has the lowest R_ct_ = 7 Ω, corroborating our low overpotential and low Tafel-slope results that assign Ni_5_Fe-CNT the highest electrocatalytic OER activity. RuO_2_ has an R_ct_ of 39 Ω, which matches the literature (RuO_2_ R_ct_ = 40 Ω [78]).

**Figure 8:**
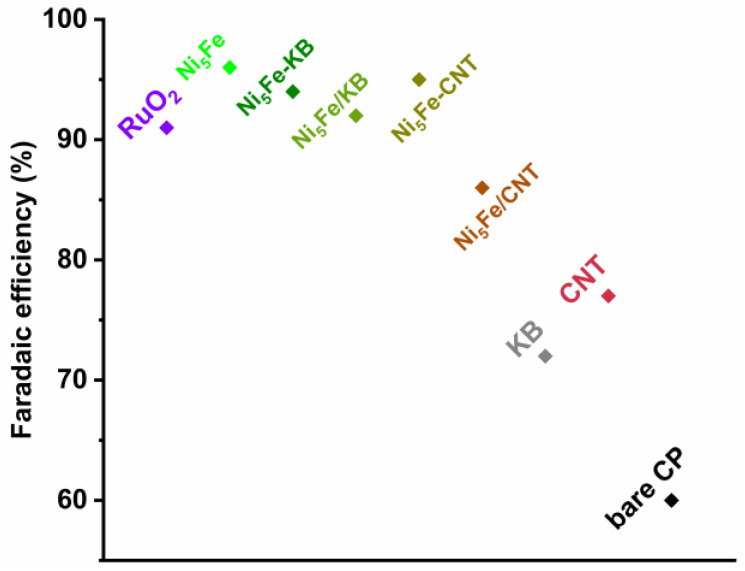
Faradaic efficiencies of RuO_2_, Ni_5_Fe, Ni_5_Fe-KB, Ni_5_Fe/KB, Ni_5_Fe-CNT, Ni_5_Fe/CNT, KB, CNT (with correction, see Table S7), and bare carbon paper (CP) (without correction, see Table S7).

Since the mixed-metal samples with the highest amount of iron (Ni_5_Fe) have the best OER performance, they were further characterized with their faradaic efficiencies (FE) (using the method of Galán-Mascarós et al. [79]; see Section 3.4 for details). Ni_5_Fe-BTC achieved the highest FE with 96%, closely followed by Ni_5_Fe-CNT with FE = 95% and Ni_5_Fe-KB with FE = 94% (Supplementary Table S7). The postsynthetically mixed Ni_5_Fe/KB reached an FE of 92%, and Ni_5_Fe/CNT an FE of 86%. All samples, with the exception of Ni_5_Fe/CNT (FE = 86%), attained a higher FE than RuO_2_ (FE = 91%) (Lit. FE ~93% [80]). The neat carbon materials KB achieved an FE of 72%, and CNT an FE of 77%. A graphical illustration of the FEs is displayed in Figure 8.

**Figure 9:**
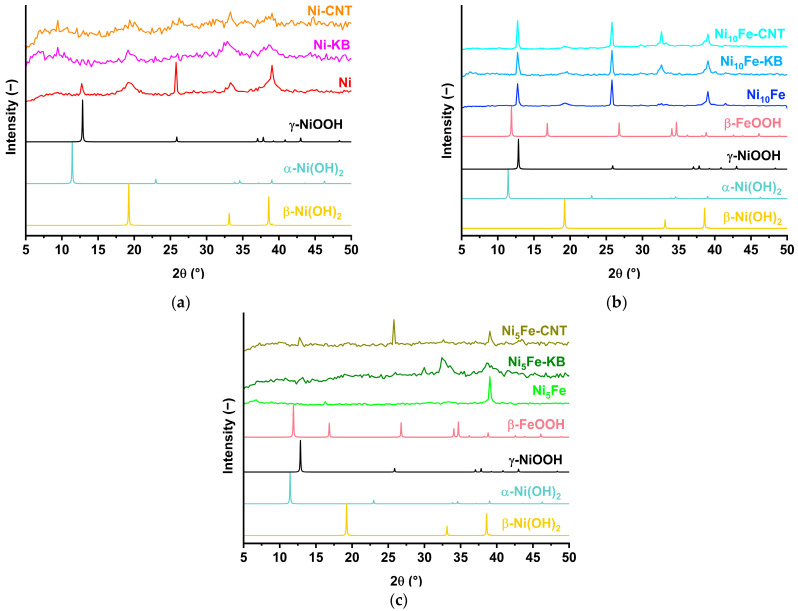
PXRD patterns of (**a**) Ni-MOF, (**b**) Ni_10_Fe, (**c**) Ni_5_Fe, and their carbon composites after 24 h in 1 mol/L KOH, and the simulated diffractograms of α-Ni(OH)_2_ (COD: 9012316), β-Ni(OH)_2_ (ICSD: 169978), γ-NiOOH (COD: 9012319), and β-FeOOH (ICSD: 167358).

As mentioned in the introduction and at the beginning of this electrochemical section, MOFs typically act as pre-catalysts that form the active phase (often metal (oxy)hydroxides) in the alkaline electrolyte and under the electrochemical reaction conditions due to their instability in the electrolyte medium [12,19,20,21]. The behavior of the MOFs and their carbon composites in the alkaline electrolyte was investigated through soaking the materials in 1 mol/L KOH for 24 h. In the PXRD patterns of all samples (Figure 9), a change from the MOF structure to their metal (oxy)hydroxides can be observed through reflexes, which fit to β-Ni(OH)_2_ (ICSD: 169978) and/or γ-NiOOH (COD: 9012319). Similar structural changes and loss of crystallinity of Ni_x_Fe-MOFs and their composites were already observed in the reported literature [14,20,28,35]. Noteworthy, the crystallinity of the hydrolysis products varies strongly, from largely amorphous for the Ni-MOF-carbon composites and Ni_5_Fe-KB to well crystalline for the Ni_10_Fe-MOF and its carbon composites. In a 12 h chronopotentiometric measurement, good stability of all derived electrocatalysts and RuO_2_ was shown.

In order to assess the advantage of using sacrificial mixed-metal MOFs to derive mixed-metal hydroxide composites, we also compared the activity of the electrocatalyst derived from a physical mixture of Ni(OH)_2_ and FeOOH (molar Ni:Fe ratio 10:1 or 5:1).

Commercially available Ni(OH)_2_ and freshly precipitated FeOOH (from 40 mg Fe(NO_3_)_3_·9H_2_O added to 20 mL of a 1 mol/L KOH solution) were then mixed in a 10:1 or 5:1 (Ni:Fe) molar ratio. Together with a neat Ni(OH)_2_ sample, the obtained mixed-metal hydroxide mixtures were used for the electrochemical measurements. From the LSV plots before and after 1000 CVs (Supplementary Figure S32a), these metal hydroxides yielded the following: for Ni(OH)_2_:FeOOH 10:1, *η*_10_ *=* 423 mV before and 427 mV after activation; for Ni(OH)_2_:FeOOH 5:1, *η*_10_ *=* 410 mV before and 411 mV after activation. Yet, these overpotentials are significantly higher than those obtained from the catalyst derived from Ni_10_Fe-MOF (311 mV before, 320 mV after) and Ni_5_Fe-MOF (296 mV before, 314 mV after). This clearly shows, once again [60], that the use of mixed-metal MOFs provides for an advantageous intimate metal mixture on the nanoscale in the derived hydroxides. Ni(OH)_2_ even reached, before activation, only a current density of 8 mA/cm^2^ with *η*_8_ *=* 470 mV, and after activation, the current density did not come close to 10 mA/cm^2^ anymore (Figure S32a,b, Supplementary Table S8). Whereas the catalyst from the Ni-MOF had *η*_10_ *=* 372 mV before and 379 mV after activation. Similar to the MOF samples, a higher iron content in the metal hydroxide samples led to a smaller overpotential and a smaller Tafel slope value *b*: for Ni(OH)_2_, *b* = 140 mV/dec; for Ni(OH)_2_:FeOOH 10:1, *b* = 130 mV/dec; for Ni(OH)_2_:FeOOH 5:1, *b* = 119 mV/dec (Figure S32c–f, Supplementary Table S8, Section S8.5 Metal hydroxides).

## 3. Materials and Methods

### 3.1. Materials

The chemicals used were obtained from commercial sources and no further purification was carried out. The carbon nanotubes were multi-walled carbon nanotubes (OD: 10–30° nm; L: 5–15° µm; 95+%) from IOLITEC Ionic Liquids Technologies GmbH (Heilbronn, Germany). Ketjenblack EC 600 JD was purchased from AkzoNobel, Amsterdam, The Netherlands. The MOFs Ni-BTC, Ni_10_Fe, Ni_5_Fe, and their carbon composites were synthesized according to our previous work on the Ni_x_Co-MOF with some modifications [35].

### 3.2. Synthesis of the In Situ MOF-Composites

For the Ni-BTC sample, 378 mg (1.3 mmol) Ni(NO_3_)_2_·6H_2_O, 205 mg (0.98 mmol) H_3_BTC, and 55 mg (0.67 mmol) 2-MeImH were dissolved in 15 mL of DMF at RT and stirred for 30 min [40]. For the in situ Ni-KB or CNT samples, the same amounts were used, and 10 wt% KB or CNT with respect to the educts for the MOF were added.

For the Ni_10_Fe sample, 349 mg (1.2 mmol) Ni(NO_3_)_2_·6H_2_O, 48 mg (0.11 mmol) Fe(NO_3_)_3_·9H_2_O, 205 mg (0.98 mmol) H_3_BTC, and 55 mg (0.67 mmol) 2-MeImH were dissolved in 15 mL of DMF at RT and stirred for 30 min. For the in situ Ni_10_Fe-KB or -CNT samples, the same amounts were used, and 10 wt% CNT or KB in regards to the weighted educts for the MOF were added.

For the Ni_5_Fe sample, 314 mg (1.08 mmol) Ni(NO_3_)_2_·6H_2_O, 89 mg (0.22 mmol) Fe(NO_3_)_3_·9H_2_O, 205 mg (0.98 mmol) H_3_BTC, and 55 mg (0.67 mmol) 2-MeImH were dissolved in 15 mL of DMF at RT and stirred for 30 min. For the in situ Ni_5_Fe-KB or -CNT samples, the same amounts were used, and 10 wt% CNT or KB in regards to the weighted educts for the MOF were added.

The prepared solutions were transferred into a Teflon-lined stainless-steel autoclave and heated to 170 °C for 48 h. The resulting dark green (Ni-MOF), dark olive-green (Ni_10_Fe, Ni_5_Fe), and black (Ni_x_Fe-KB or -CNT) precipitates were separated by centrifugation (15 min, 6000 rpm). The precipitates were washed once with DMF (15 mL, 5 h) and twice with EtOH (15 mL each, 10 h) and centrifuged again (15 min, 6000 rpm). The products were dried overnight in a vacuum drying cabinet at 120 °C and a reduced pressure of less than 50 mbar.

Yields:

Ni-MOF: 277 mg; Ni-KB: 353 mg; Ni-CNT: 318 mg

Ni_10_Fe: 320 mg; Ni_10_Fe-KB: 434 mg; Ni_10_Fe-CNT: 358 mg

Ni_5_Fe: 366 mg; Ni_5_Fe-KB: 430 mg; Ni_5_Fe-CNT: 472 mg

For the postsynthetically mixed MOF/carbon composites, the synthesized neat MOFs were physically mixed with 10 wt% KB or CNT in regards to the weighted neat MOF amount. Specifically, 50 mg of the neat MOFs and 5.5 mg of the carbon material (KB or CNT) were mixed at 40 Hz for 20 min with a vortex mixer.

### 3.3. Materials Characterization

A Bruker D2 Phaser powder diffractometer (Bruker AXS, Karlsruhe, Germany) with a power of 300 W and an acceleration voltage of 30 kV at 10 mA using Cu-Kα radiation (λ = 1.5418 Å) was used for the PXRD measurements at ambient temperature. The diffractograms were gathered on a low background flat silicon sample holder and evaluated with the Match 3.11 software. The measuring range was from 5 to 50° 2θ with a scan speed of 2 s/step and a 0.057° (2θ) step size.

The FT-IR spectra were measured in KBr mode on a Bruker TENSOR 37 IR spectrometer (Bruker, Rosenheim, Germany) in the range of 4000–400 cm^–1^.

Nitrogen sorption isotherms were recorded with a Nova 4000e from Quantachrome (Anton Paar QuantaTec, Boynton Beach, FL, USA) at 77 K. The sorption isotherms were analyzed with the NovaWin 11.03 software. Before the measurements, the samples were activated for 5 h under vacuum (<10^–2^ mbar) at 120 °C. Brunauer–Emmett–Teller (BET) surface areas were determined from the adsorption branches of the isotherms, and the pore size distributions were derived by non-local density functional theory (NLDFT) calculations based on N_2_ at 77 K on carbon with slit/cylindrical pores.

TGA was performed with a Netzsch TG 209 F3 Tarsus device (Erich NETZSCH B.V. & Co. Holding KG, Selb, Germany) equipped with an Al crucible. A heating rate of 10 K/min under a N_2_ atmosphere was applied for the measurements.

SEM images were acquired with a JEOL JSM-6510 LV QSEM advanced electron microscope (Jeol, Akishima, Japan) with a LaB_6_ cathode at 20 kV. The microscope was equipped with a Bruker Xflash 410 silicon drift detector (Bruker, Berlin, Germany) for the EDX analysis. The Au, Cu, and Zn detected in the EDX spectra can be attributed to the sputtering of the sample with gold prior to the investigation and the brass sample holder, which was utilized.

The vortex mixer used to generate the postsynthetic composites is a REAX 2000 from Heidolph (Heidolph Scientific Products GmbH, Schwabach, Germany).

### 3.4. Electrocatalytic Measurements

A three-electrode setup was used for the electrocatalytic measurements, which were recorded with a SP-50e potentiostat from BioLogic (BioLogic Science Instruments, Göttingen, Germany). The counter electrode was a Pt plate, the reference electrode was a reversible hydrogen electrode (RHE) from Gaskatel (Kassel, Germany), and the working electrode was ink-coated carbon paper. A carbon paper sheet was cut into 2 × 1 cm pieces. The electrocatalyst inks contained 2.5 mg of electrocatalyst, 0.5 mL ethanol, and 20 μL Nafion (5 wt.%) and were sonicated for 30 min. One hundred μL of ink was drop-cast onto a defined 1 × 1 cm part of the prepared carbon paper piece (geometric area of 1 cm^2^), which resulted in a catalyst loading of 0.5 mg/cm^2^. The ink-coated part of the carbon paper piece was immersed in the electrolyte. The non-coated part was fixed in a clamp containing a platinum plate contacted to a gold pin, which was connected with clamps to the potentiostat (see three-electrode setup in Supplementary Figure S33). The loading of the electrocatalyst onto the carbon paper was additionally checked with the weight gain of the carbon paper piece, which also showed a catalyst loading of 0.5 mg_sample_/g_carbon paper_.

SEM images of the carbon paper with and without ink-coating before and after the electrochemical measurements for the mixed-metal samples in Supplementary Figures S18–S23 show good adherence of the materials to the carbon paper substrate. A 1 mol/L KOH solution was utilized as the electrolyte for all electrocatalytic measurements, which had been purged with N_2_ for 10 min before the electrocatalytic experiments. The samples were activated by cycling the working electrode between 1.0 V and 1.7 V vs. RHE at a scan rate of 100 mV/s for 10 cycles. The LSV polarization curves were measured in a potential range of 1.0 to 1.7 V vs. RHE at a scan rate of 5 mV/s. The cycling stability was measured by comparing activated LSV curves before and after 1000 CV cycles between 1–1.7 V with a scan rate of 100 mV/s. EIS data were collected in a frequency range of 0.01 Hz to 10 kHz at a potential of 1.5 V vs. RHE. Chronopotentiometry was conducted following the above-mentioned electrocatalytic measurements. The current density was held at 10 mA/cm^2^ for 12 h. The overpotential *η* was calculated according to: *η* = E − E° (1.23 V) − IR. The OER performance of the MOF samples and their composites were compared with commercial RuO_2_ (Sigma-Aldrich, St. Louis, MO, USA), multi-walled CNTs (OD: 10–30 nm; L: 5–15 µm; 95+%; IOLITEC Ionic Liquids Technologies GmbH, Heilbronn, Germany), and KB (AkzoNobel, The Netherlands). At least two measurements were done for each sample to determine the overpotentials, Tafel slopes, and R_ct_ in order to reduce experimental contingency error, and the averaged results were displayed in the figures and tables. All electrochemical measurements were automatically corrected with the IR.

To determine the FE, the method of Galán-Mascarós et al. was followed [79]. A chronopotentiometric measurement was conducted at a fixed current density of 10 mA/cm^2^, while an Ocean Optics NeoFOX sensor system (Ocean Optics B. V., Ostfildern, Germany) coupled with a FOSPOR probe (Ocean Optics B. V., Ostfildern, Germany) was used to record the O_2_ level in the headspace of the electrochemical cell. The calibration of the FOSPOR probe was done through a two-point calibration with N_2_ atmosphere (0% O_2_) as one calibration point and ambient air (21% O_2_) as the other calibration point. Before the chronopotentiometric test, the electrolyte underwent thorough deaeration by continuous purging with N_2_ for at least 1 h. To calculate the molar amount of O_2_ evolved during the electrochemical measurement, Equation (5) was used, which considers ideal gas behavior:

*n*_O2,exp_ = (% O_2,det_ × *P*_total_ × *V*_gas_/*R* × *T*)/100 (5)

where % O_2,det_ is the detected percentage of oxygen measured by the FOSPOR probe (corrected by the % O_2,det_ from a measurement without any applied current to consider the oxygen leakage from ambient air); *P*_total_ is 1 atm; *V*_gas_ (L) is the developed gas volume at atmospheric pressure; *R* is the universal gas constant (0.082 atm × L/K × mol); and *T* is the absolute temperature (293 K). The theoretically generated faradaic oxygen is obtained through Equation (6):

*n*_O2_,_far_ = *Q*/*n*_e_ × *F* (6)

where *Q* (measured in Coulombs, C) stands for the total electric charge passed through the system; *n*_e_ represents the needed molar amount of electrons to produce one mol of O_2_ (equals to 4), and *F* is the Faraday constant (96,485 C/mol). The FE (expressed in percentage) is determined by Equation (7):

FE = 100 × *n*_O2,exp_/*n*_O2_,_far_ (7)

## 4. Conclusions

In this work, a series of Ni-, mixed-metal Ni_x_Fe-MOFs and their composites with the carbon materials ketjenblack (KB) and carbon nanotubes (CNT) were synthesized both in situ through a simple one-pot solvothermal reaction containing the MOF precursors and the carbon material, or postsynthetically mixed from the prepared MOF and carbon material. The in situ synthesized MOF-carbon composites were fully characterized concerning the successful MOF formation in the presence of carbon. As seen before, in the alkaline electrochemical environment, the MOFs act as precursors to the actual metal (oxide)hydroxide catalysts and provide an intimate mixture of the mixed metals and the carbon material on the nanoscale in the derived metal hydroxide catalyst material when compared with a physical mixture of Ni(OH)_2_ and FeOOH. The carbon materials KB and CNT increase the low electrical conductivity of the derived metal (oxide)hydroxide electrocatalysts. All of the tested Ni_10_Fe- and Ni_5_Fe-MOF-carbon electrocatalysts outperformed the RuO_2_ benchmark, whereas the nickel-only Ni-BTC and its carbon composite were similar to or worse than RuO_2_. Thus, with an increasing amount of Fe, the electrocatalytic activity regarding the OER could be improved. Concerning the assessed comparison of the in situ and postsynthetic preparation of the MOF mixtures with KB and CNT, for KB the postsynthetic materials and for CNT the in situ ones have the lower overpotential, while the Tafel slopes, charge transfer resistance (R_ct_) and faradaic efficiency are rather similar. Evidently, no clear advantage can be seen for one type of MOF-carbon composite formation over the other, although a large number of experiments were carried out. The similar electrocatalytic behavior of the in situ and postsynthetic MOF composites may derive from the MOF precursor function and the inherent metal hydroxide formation in the 1 mol/L KOH electrolyte, which alleviates differences in the initial MOF-carbon composites.

At the same time, the addition of Fe to neat Ni-BTC had a significant effect on reducing the overpotential, Tafel slope and charge transfer resistance. In the bimetallic NiFe-MOFs, the Fe component can promote the reversible transformation from α-/β-NiCo(OH)_2_ to β-/γ-NiCoOOH during the electrocatalytic charging/discharging according to the Bode scheme [81], and, thereby, the synergistic effect between the two metals at the nanoscale can improve the OER performance, lower reaction resistance, and enhance intrinsic activity [82].

The best performing MOF-derived electrocatalyst came from the in situ synthesized Ni_5_Fe-CNT, with an overpotential (*η*) of 301 mV (RuO_2_ *η* = 354 mV), a Tafel slope (*b*) of 58 mV/dec (RuO_2_ *b* = 91 mV/dec), a charge transfer resistance (R_ct_) of 7 Ω (RuO_2_ R_ct_= 39 Ω) and a faradaic efficiency (FE) of 95% (RuO_2_ FE = 91%). The postsynthetic materials Ni_5_Fe/KB and Ni5Fe/CNT were close behind. The higher OER activity of the Ni_5_Fe-MOF composites vs. Ni-MOF and Ni_10_Fe is clearly due to the presence and larger amount of iron. Noteworthy, the Ni:Fe ratio of 5:1 is much lower than the OER optimum of 32:1, where the maximum current density was obtained for NiO/Fe_2_O_3_ mixtures (between Ni:Fe = 64:1 and 1:1, with also neat NiO and neat Fe_2_O_3_) loaded on nickel foam [82]. Yet, for the composite of Ni_10_Fe with KB, the postsynthetic mixture performed significantly better in terms of overpotential and R_ct_ than the in situ material (314 vs. 339 mV and 17 vs. 33 Ω, respectively), which shows that the type of mixing can still present an additional improvement. Future research could take into account the effect on OER activity of in situ and postsynthetically mixed composites with a higher amount of KB or CNT, and other carbon materials could be further added to the comparison.

## Data Availability

The data presented in this study are available on request from the corresponding author.

## Supplementary Materials

https://www.mdpi.com/article/10.3390/molecules30020208/s1, Section S1: Ni-BTC structure; Section S2: Energy-dispersive X-ray spectroscopy (EDX) from scanning electron microscopy (SEM); Section S3: Scanning electron microscopy (SEM); Section S4: Powder X-ray diffraction (PXRD); Section S5: Thermogravimetric analysis (TGA); Section S6: Fourier transform infrared (FT-IR) spectroscopy; Section S7: Porosity-related parameters derived from N_2_-sorption measurements; Section S8: Electrochemical Data; Section S9: References.
