# Supplementary material for: Comparison of In Situ and Postsynthetic Formation of MOF-Carbon Composites as Electrocatalysts for the Alkaline Oxygen Evolution Reaction (OER)
Source: Molecules. 2025 Jan 7;30(2):208. doi: 10.3390/molecules30020208 (PMC11767250; doi:10.3390/molecules30020208)
Supplement: Supplementary file 1 [file molecules-30-00208-s001.zip › molecules-3385509-supplementary.pdf]

Supporting Information

for

Comparison of in situ and postsynthetic formation of MOF-carbon composites as electrocatalysts for the alkaline oxygen evolution reaction (OER)

Linda Sondermann [1], Laura Maria Voggenauer [1], Annette Vollrath [1], Till Strothmann [1] and Christoph Janiak [1,*]

[1]Institut für Anorganische Chemie und Strukturchemie, Heinrich-Heine-Universität Düsseldorf, 40225 Düsseldorf, Germany; linda.sondermann@hhu.de (L.S.), lavog108@uni-duesseldorf.de (L.V.), annette.vollrath@hhu.de (A.V.), till.strothmann@hhu.de (T.S.)

*Correspondence: janiak@uni-duesseldorf.de, Tel.: +49-211-81-12286 (C.J.)

Section S1. Ni-BTC structure

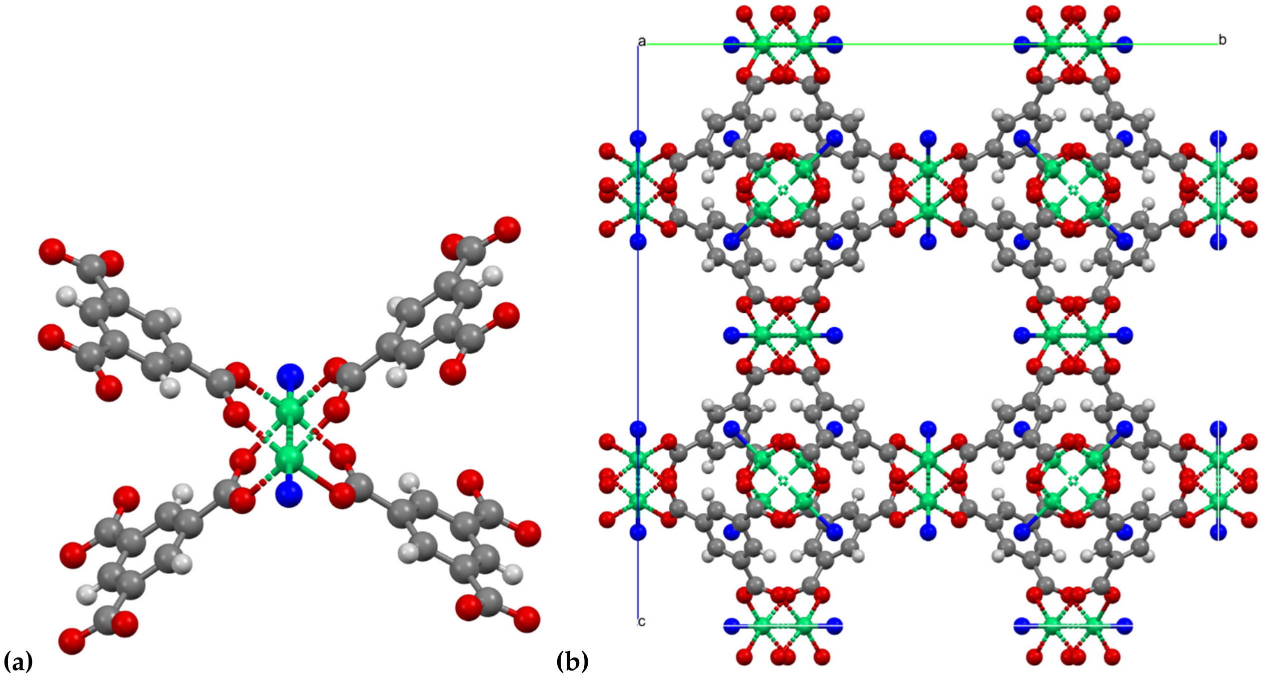

(a) **(b)**

Figure S1. (a) $\{Ni_2(BTC)_4\}$ "paddle-wheel" unit in Ni-BTC, $[Ni_3(BTC)_2(Me_2NH)_3]\cdot(DMF)_4(H_2O)_4$.
(b) The cubic unit cell packing in Ni-BTC without the crystal solvent molecules. From the disordered dimethylamine ligand on Ni only the nitrogen atom is shown. Color code: Ni green, O red, N blue, C dark gray, H light gray. The structure was drawn from the deposited cif file with CCDC no. 802889 [1].

Section S2. Energy-dispersive X-ray spectroscopy (EDX) from scanning electron microscopy (SEM)

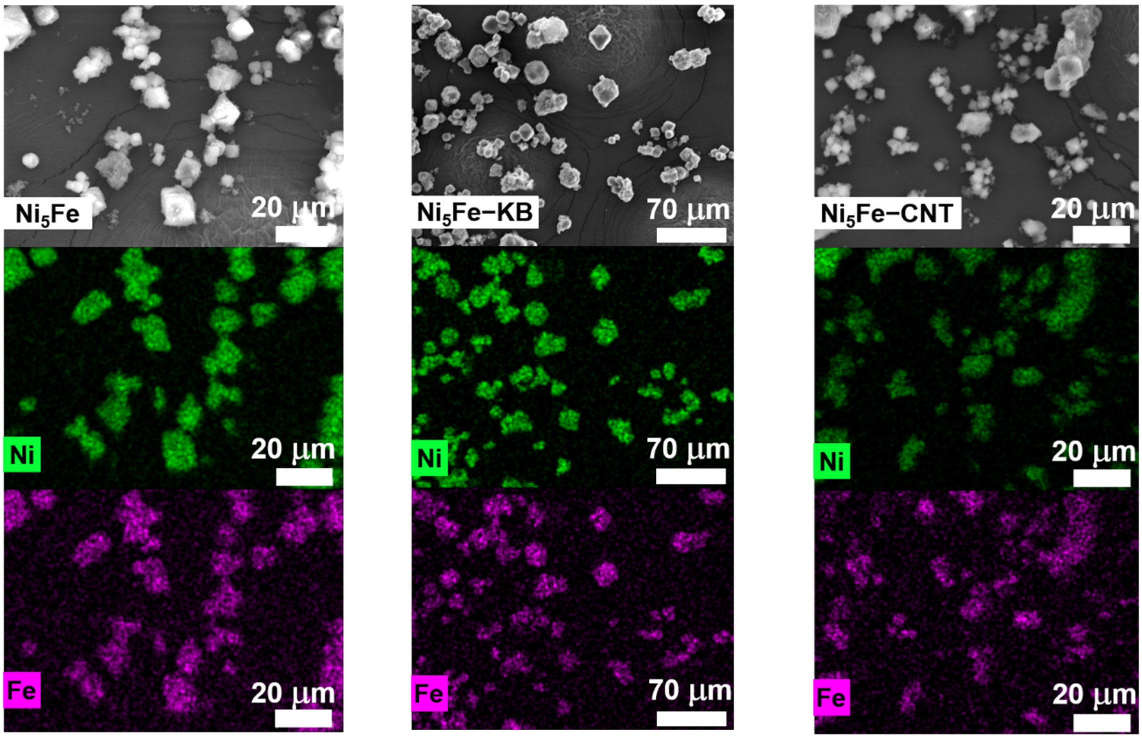

(**a**) (**b**) (**c**)

Figure S2. SEM images (first row) and EDX elemental mapping for Ni (second row) and Fe (third row) for **(a)** Ni_5Fe, **(b)** Ni_5Fe-KB and **(c)** Ni_5Fe-CNT. Further SEM images and the SEM-EDX spectra are displayed in Fig. 2 and S3 to S9. Note the larger scale for the KB composites.

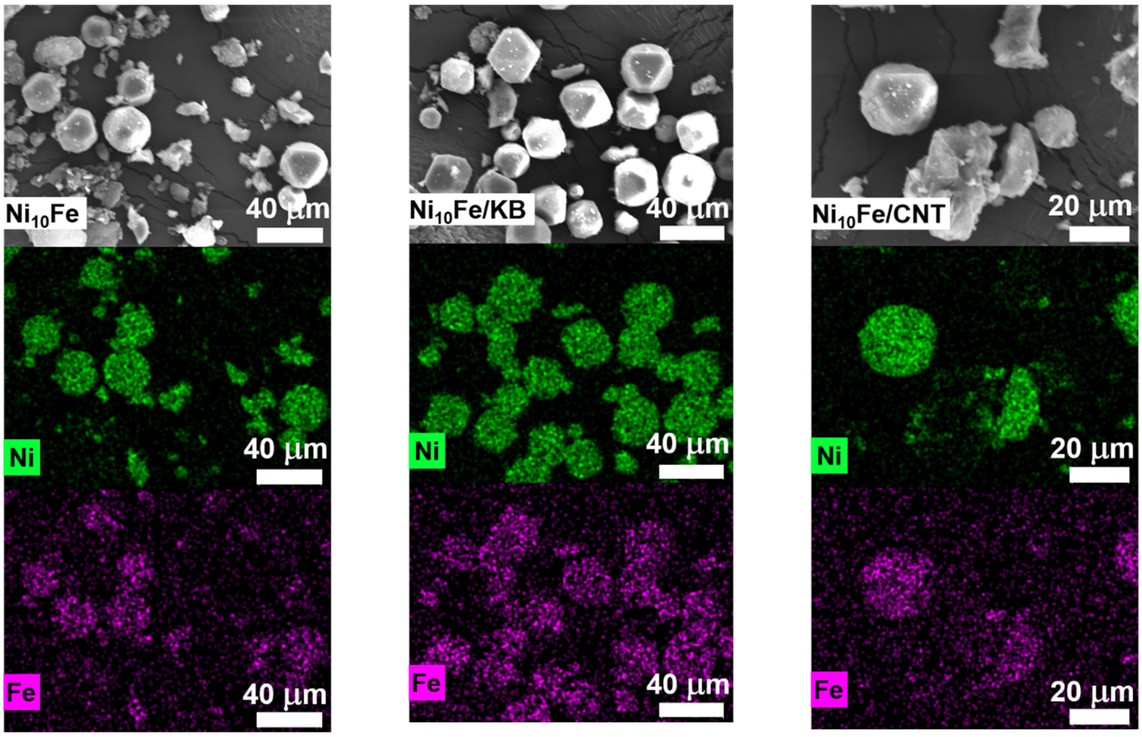

(**a**) (**b**) (**c**)

Figure S3. SEM images (first row) and EDX elemental mapping for Ni (second row) and Fe (third row) for **(a)** $Ni_{10}Fe$, **(b)** $Ni_{10}Fe$/KB and **(c)** $Ni_{10}Fe$/CNT. Further SEM images and the SEM-EDX spectra are displayed in Fig. 2 and S2 to S9. Note the smaller scale for the CNT composites.

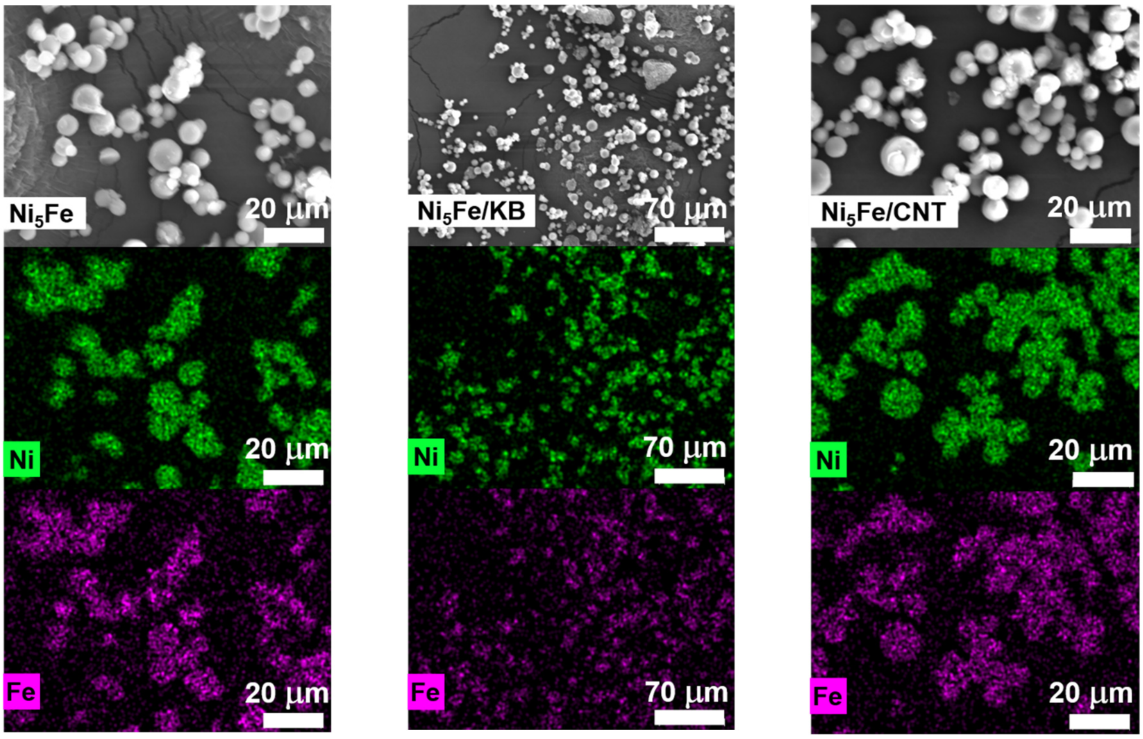

(**a**) (**b**) (**c**)

Figure S4. SEM images (first row) and EDX elemental mapping for Ni (second row) and Fe (third row) for **(a)** Ni_5Fe, **(b)** Ni_5Fe/KB and **(c)** Ni_5Fe/CNT. Further SEM images and the SEM-EDX spectra are displayed in Fig. 2 and S2 to S9. Note the larger scale for the KB composites.

Table S1. SEM-EDX results of the molar Ni:Fe metal ratios.

Sample [1]	theor. from synthesis	SEM-EDX [1]
**$Ni_{10}Fe$**	10:1	11:1, 10:1 [2]
**$Ni_{10}Fe$-KB**	10:1	11:1
**$Ni_{10}Fe$-CNT**	10:1	10:1
**Ni_5Fe**	5:1	5:1, 4:1 [2]
**Ni_5Fe-KB**	5:1	5:1
**Ni_5Fe-CNT**	5:1	5:1
**$Ni_{10}Fe/KB$**	10:1	9:1
**$Ni_{10}Fe/CNT$**	10:1	9:1
**Ni_5Fe/KB**	5:1	4:1
**Ni_5Fe/CNT**	5:1	4:1

[1] EDX is a surface-weighted spectroscopy, see text. [2] Measurements from two different samples which shows the variance in the sample preparation together with the EDX measurement.

With SEM-EDX (Table S1, Figure 2, Figure S2 to S4) elements of the samples can be quantified. In the EDX spectra (Figure S5 and S6) C, O, Ni and Fe were shown to be present. EDX is a surface-weighted method and its emitted X-rays give a 1–2 µm depth analysis. Furthermore, due to stronger matrix effects EDX as an X-ray spectroscopy requires standards for peak identification and quantification.

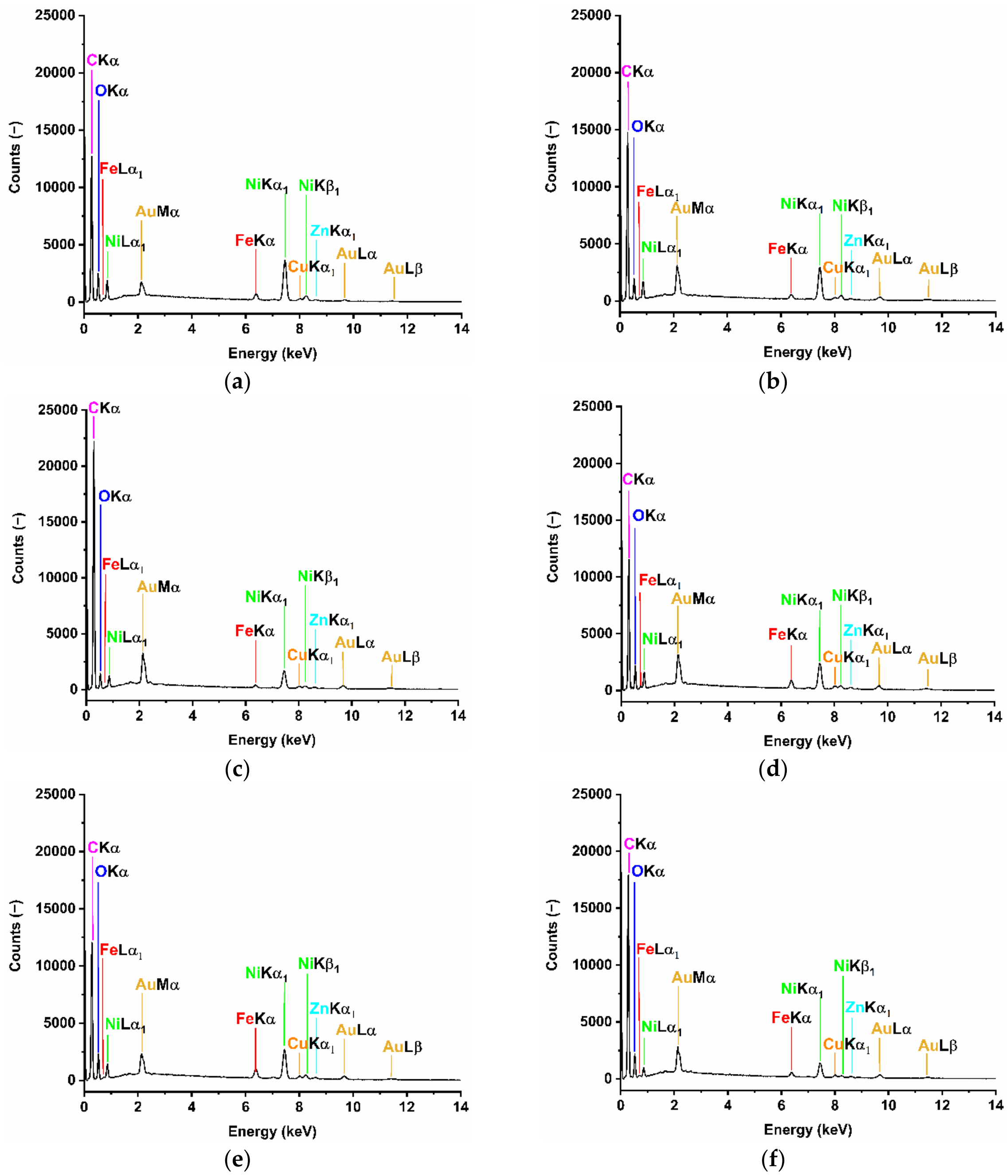

Figure S5. SEM-EDX spectra of **(a)** $Ni_{10}Fe$, **(b)** $Ni_{10}Fe$-KB, **(c)** $Ni_{10}Fe$-CNT, **(d)** Ni_5Fe, **(e)** Ni_5Fe-KB, and **(f)** Ni_5Fe-CNT. The Cu, Zn and Au signals in the EDX spectra can be attributed to the brass sample holder and the sputtering of the sample with gold before the measurement.

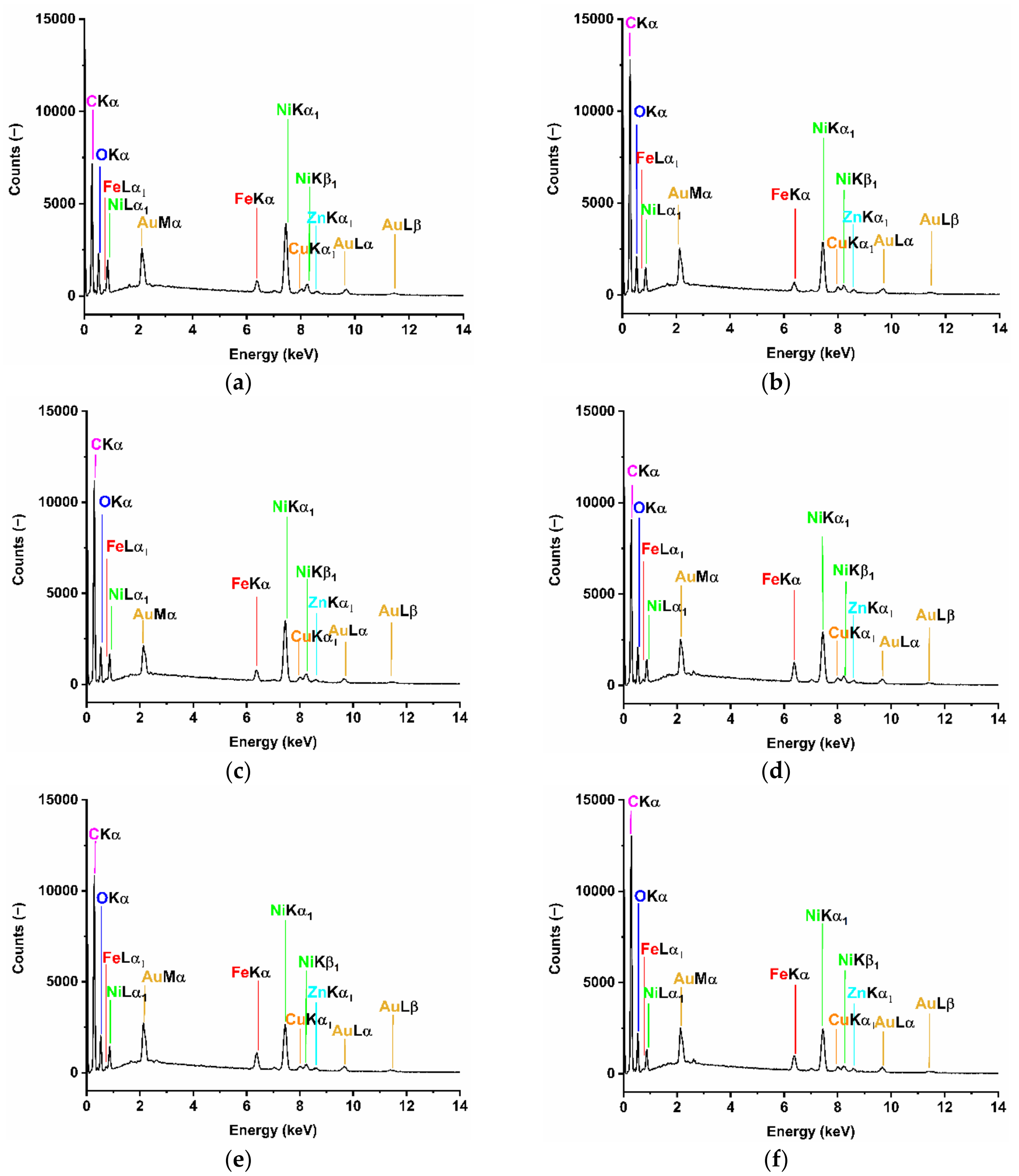

Figure S6. SEM-EDX spectra of **(a)** $Ni_{10}Fe$, **(b)** $Ni_{10}Fe$/KB, **(c)** $Ni_{10}Fe$/CNT, **(d)** Ni_5Fe, **(e)** Ni_5Fe/KB and **(f)** Ni_5Fe/CNT. The Cu, Zn and Au signals in the EDX spectra can be attributed to the brass sample holder and the sputtering of the sample with gold before the measurement.

Section S3. Scanning electron microscopy (SEM)

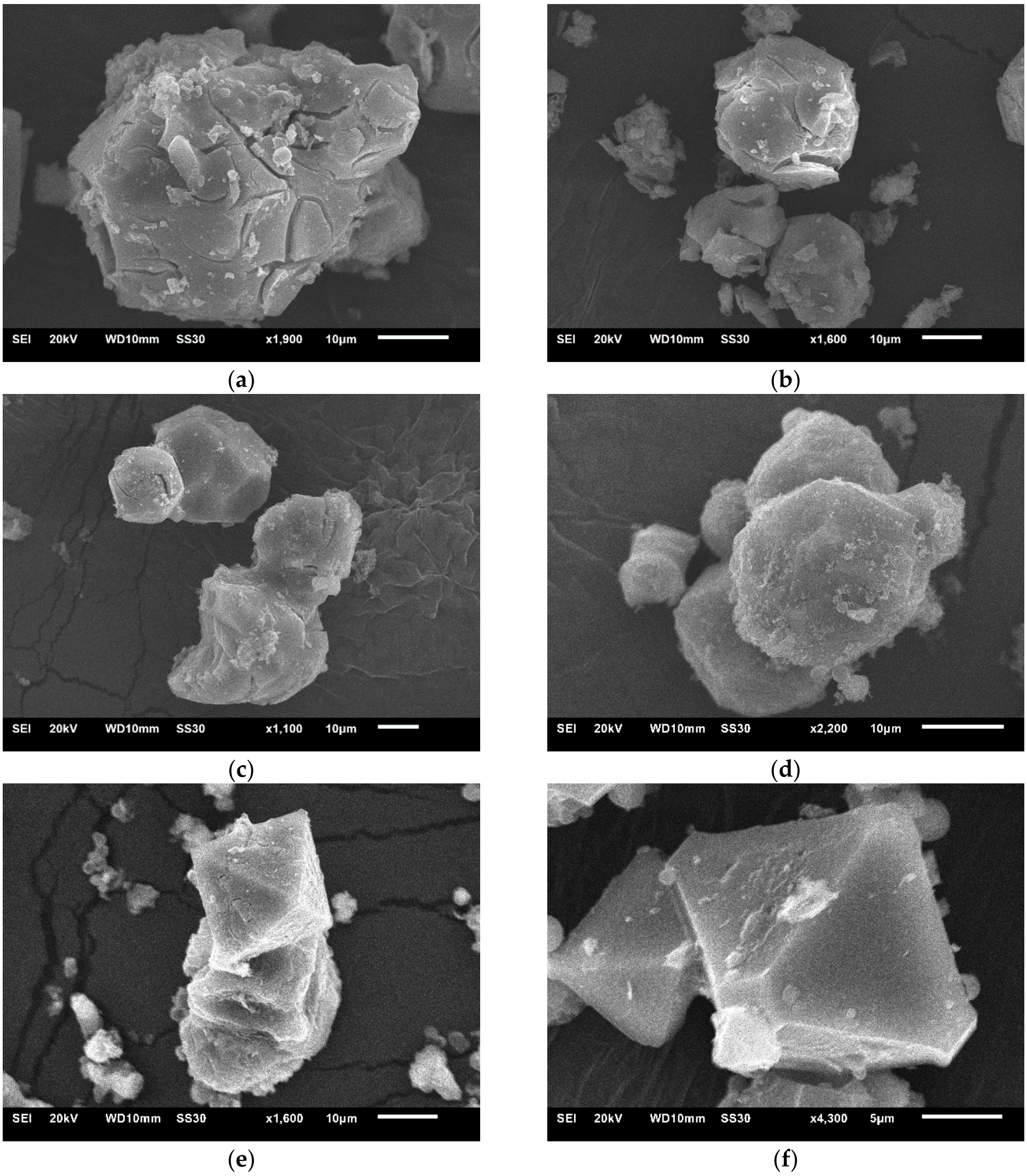

Figure S7. SEM images of **(a,b)** Ni-MOF, **(c,d)** Ni-KB and **(e,f)** Ni-CNT.

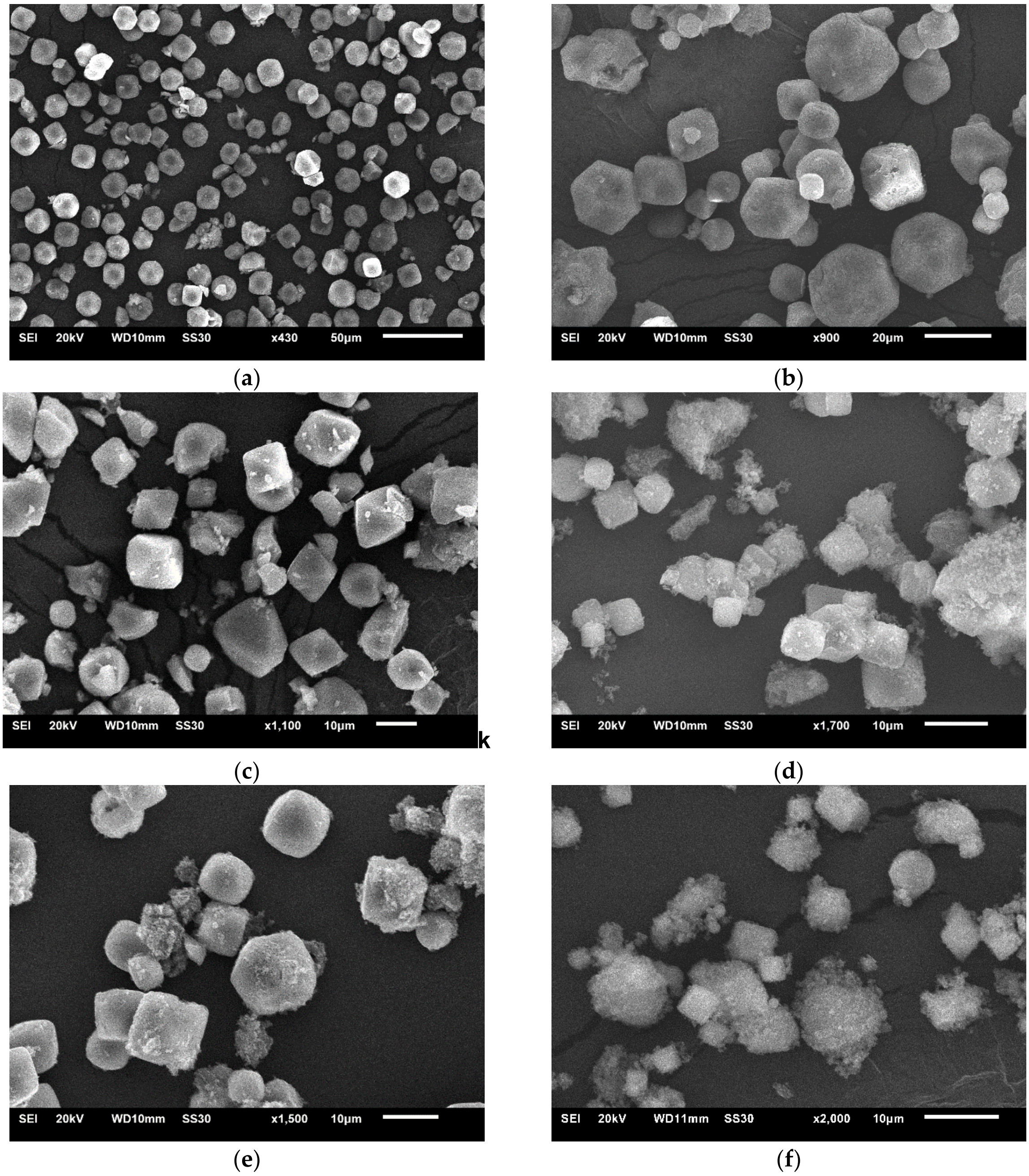

Figure S8. SEM images of **(a)** $Ni_{10}Fe$, **(b)** $Ni_{10}Fe$-KB, **(c)** $Ni_{10}Fe$-CNT, **(d)** Ni_5Fe, **(e)** Ni_5Fe-KB and **(f)** Ni_5Fe-CNT.

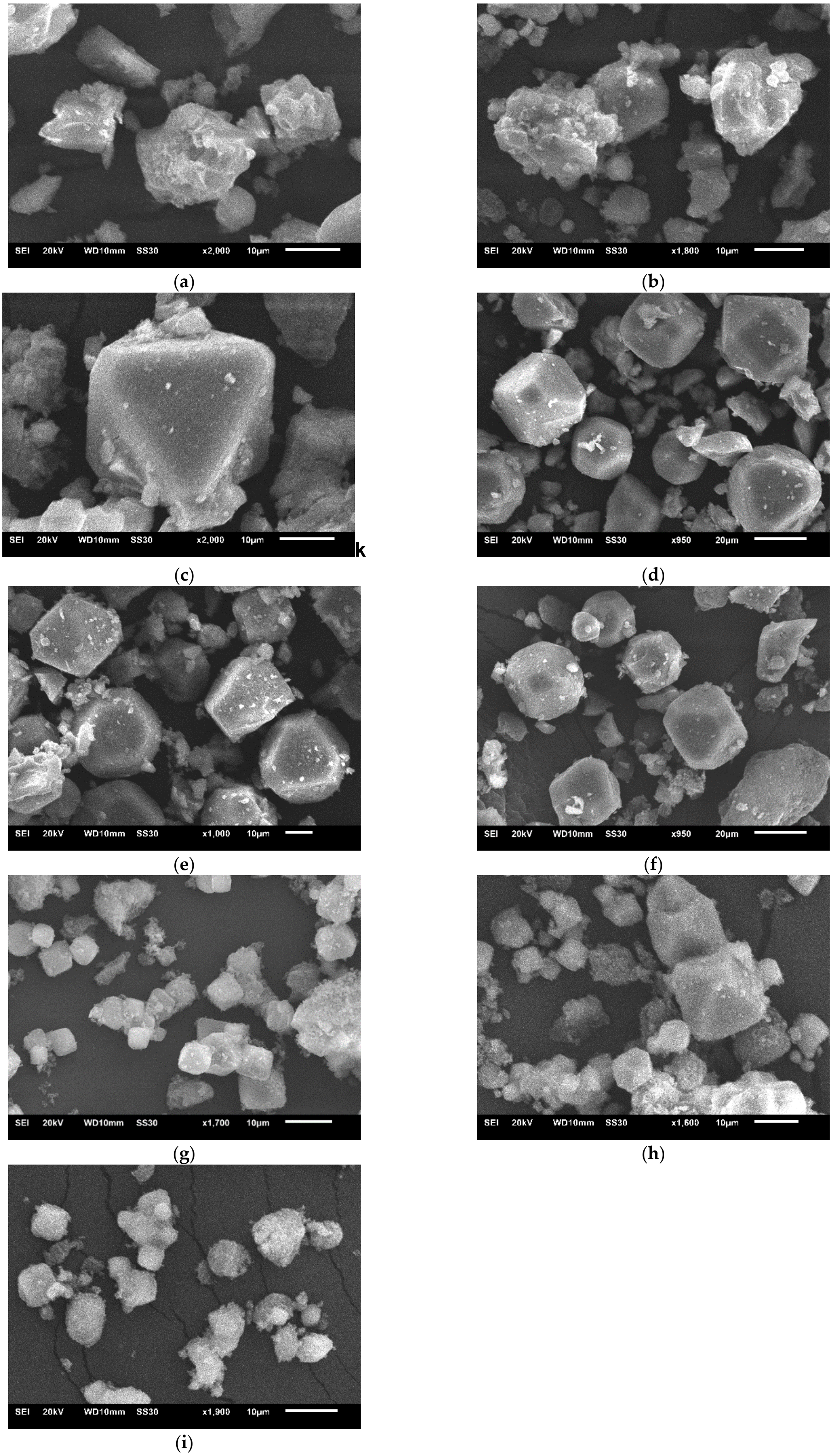

Figure S9. SEM images of **(a)** Ni-MOF, **(b)** Ni/KB, **(c)** Ni/CNT, **(d)** $Ni_{10}Fe$, **(e)** $Ni_{10}Fe$/KB, **(f)** $Ni_{10}Fe$/CNT, **(g)** Ni_5Fe, **(h)** Ni_5Fe/KB and (i) Ni_5Fe/CNT.

Section S4. Powder X-ray diffraction (PXRD)

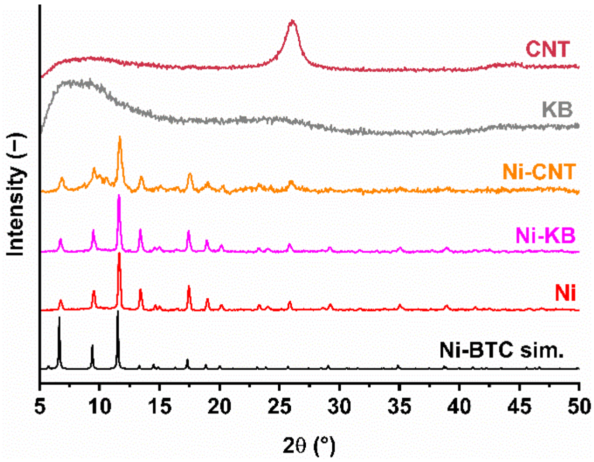

(a)

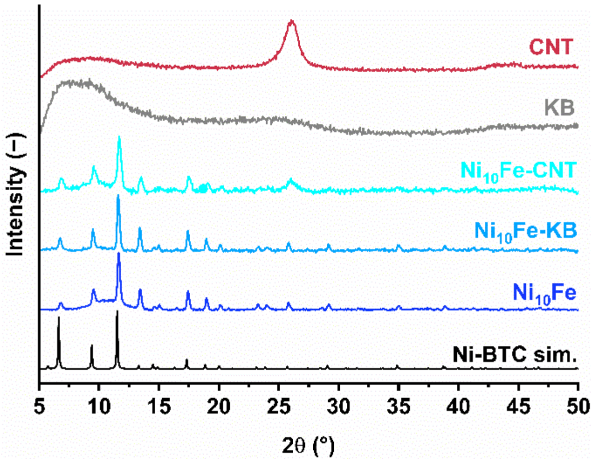

(b)

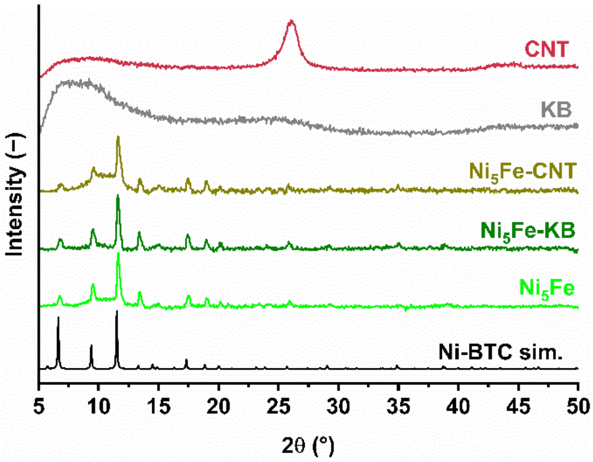

(c)

Figure S10. PXRD patterns of neat MOFs and their in situ composites together with the experimental diffractograms for CNT, KB and the simulated one for Ni-BTC (CCDC Nr. 802889). **(a)** Ni-MOF, Ni-KB, Ni-CNT, **(b)** $Ni_{10}Fe$, $Ni_{10}Fe$-KB, $Ni_{10}Fe$-CNT, **(c)** Ni_5Fe, Ni_5Fe-KB, Ni_5Fe-CNT. The low intensity of reflections at $2\theta < 7°$ is due to strong broadening of the beam spot on the flat sample holder such that only a fraction of the diffracted radiation reaches the detector.

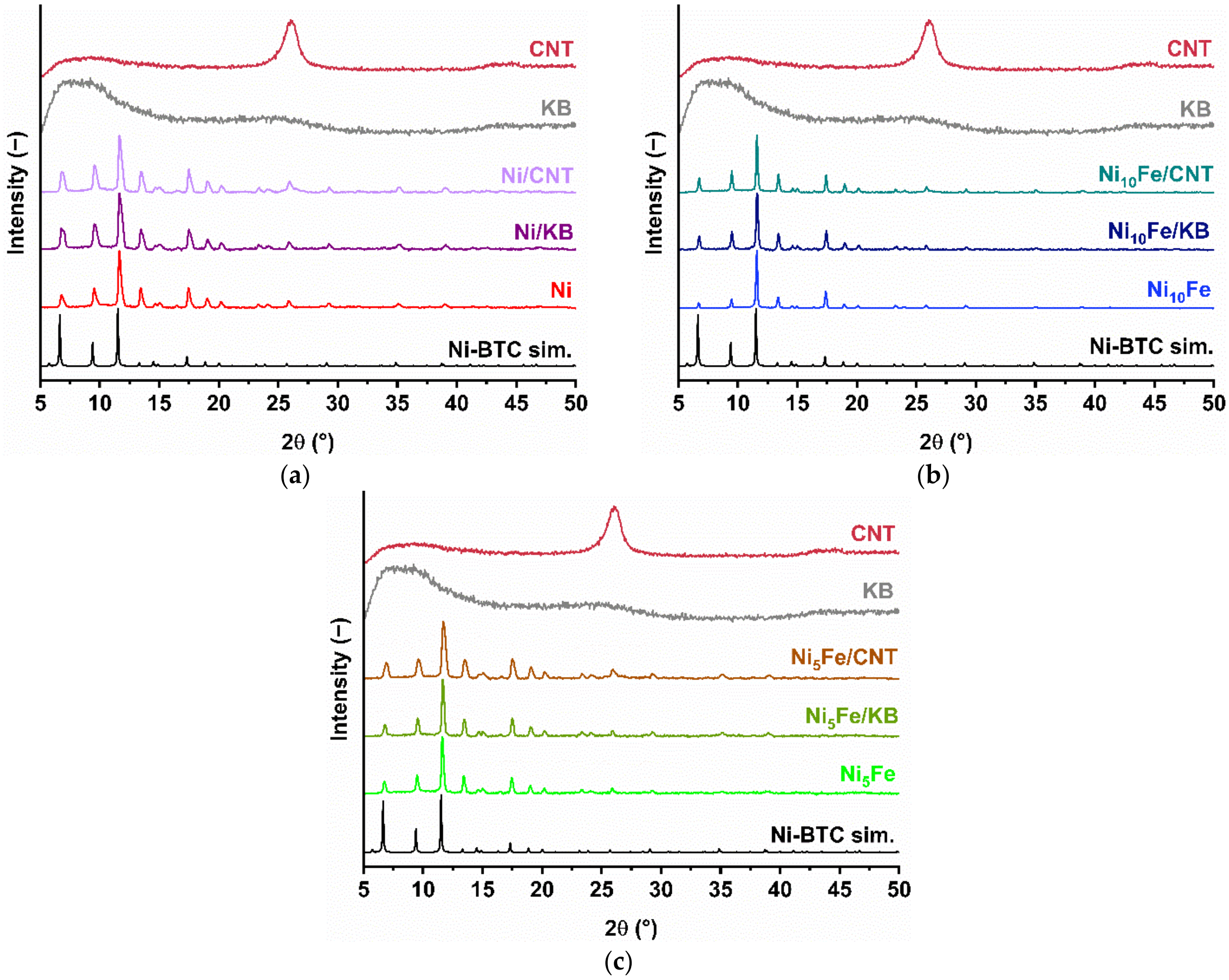

Figure S11. PXRD patterns of neat MOFs and their postsynthetic composites together with the experimental diffractograms for CNT, KB and the simulated one for Ni-BTC (CCDC Nr. 802889). **(a)** Ni-MOF, Ni/KB, Ni/CNT, **(b)** $Ni_{10}Fe$, $Ni_{10}Fe$/KB, $Ni_{10}Fe$/CNT, **(c)** Ni_5Fe, Ni_5Fe/KB, Ni_5Fe/CNT. The low intensity of reflections at $2\theta < 7°$ is due to strong broadening of the beam spot on the flat sample holder such that only a fraction of the diffracted radiation reaches the detector.

Section S5. Thermogravimetric analysis (TGA)

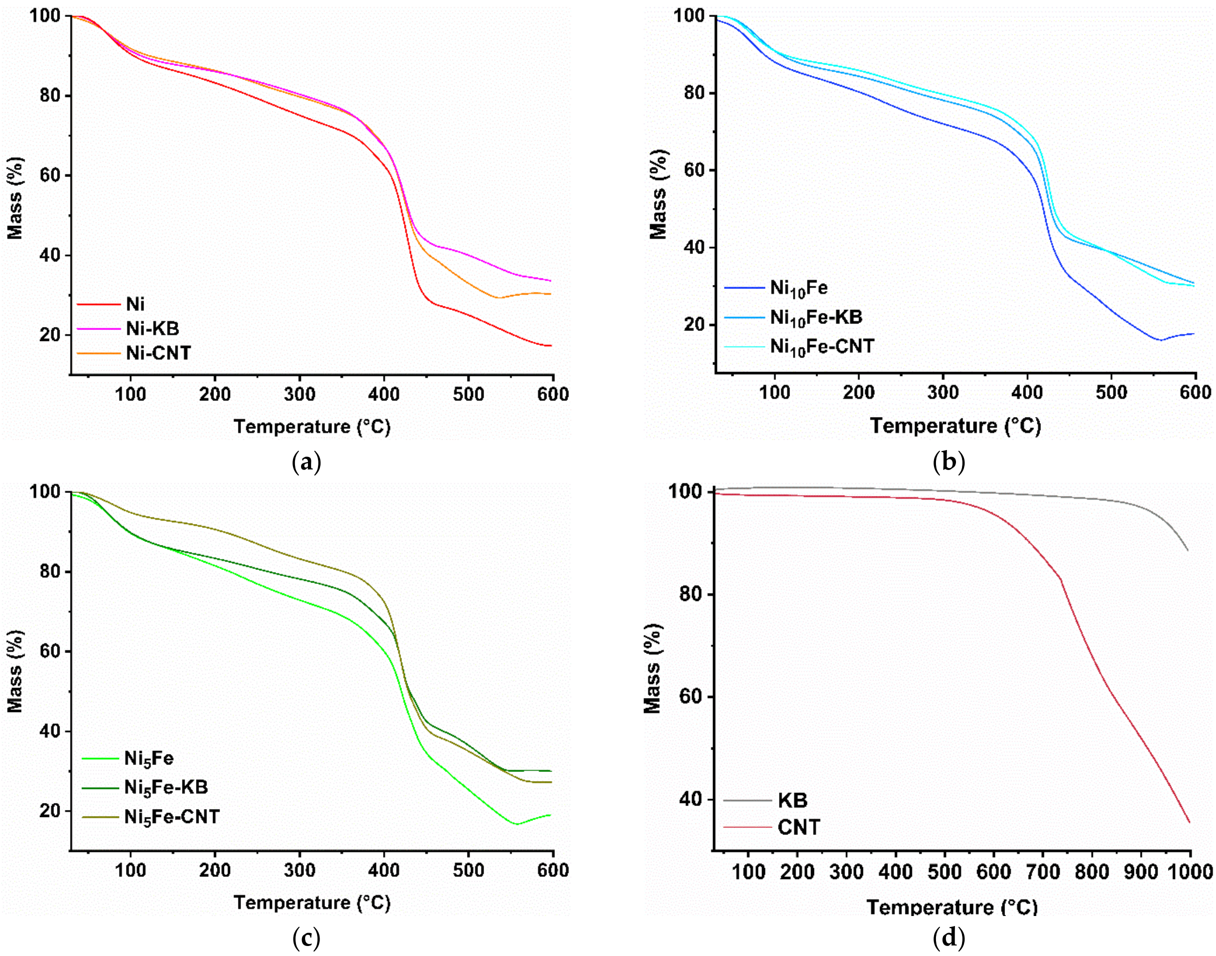

Figure S12. TGA curves neat MOFs and their in situ composites. (a) Ni, Ni-KB, Ni-CNT, **(b)** $Ni_{10}Fe$, $Ni_{10}Fe$-KB, $Ni_{10}Fe$-CNT, **(c)** Ni_5Fe, Ni_5Fe-KB, Ni_5Fe-CNT, **(d)** KB and CNT. For the TGA, the samples were used as-synthesized and had not been activated. A heating rate of 10 K/min under a N_2 atmosphere was used.

Table S2. Residual mass of pristine MOFs, their composites and the resulting carbon material amount.

Sample	Residual mass composite (wt%) [1]	Residual mass MOF (wt%) [1,2]	Carbon material amount (wt%) [3]	
			KB	CNT
**Ni-MOF**	—	17	—	—
**Ni-KB**	34	—	17	—
**Ni-CNT**	30	—	—	13
**$Ni_{10}Fe$**	—	18	—	—
**$Ni_{10}Fe$-KB**	31	—	13	—
**$Ni_{10}Fe$-CNT**	30	—	—	12
**Ni_5Fe**	—	19	—	—
**Ni_5Fe-KB**	30	—	11	—
**Ni_5Fe-CNT**	27	—	—	8

[1] At 600 °C under a N_2 atmosphere.

[2] The residual MOF mass of 17-19% at 600 °C is near the theoretical Ni mass of 16.2% for Ni-BTC of formula $[Ni_3(BTC)_2(Me_2NH)_3]\cdot(DMF)_4(H_2O)_4$ (M = 1090.02 g mol^{-1}). If part of the DMF and H_2O crystal solvent had been lost during handling before the TGA, then the residual Ni mass will be higher. For Ni-BTC of formula $[Ni_3(BTC)_2(Me_2NH)_3]\cdot(DMF)_4$ the residual Ni mass would be 17.3%. The residual mass would also be higher if in part NiO would be formed with the oxygen atom from the carboxylate donor groups. From $[Ni_3(BTC)_2(Me_2NH)_3]\cdot(DMF)_4(H_2O)_4$ the theoretical NiO mass would be 20.6%.

[3] The mass percentage of carbon material can be obtained via subtraction of the residual mass of the neat MOFs from the residual mass of their composites (Equation S1, Table S2, SI):

$$m_{\text{carbon material}}\ (\%) = m_{\text{composite}}\ (\%) - m_{\text{MOF}}\ (\%) \qquad \text{(S1)}$$

The theoretical amounts of KB and CNT from the starting materials should have been 10 wt%.

Section S6. Fourier transform infrared (FT-IR) spectroscopy

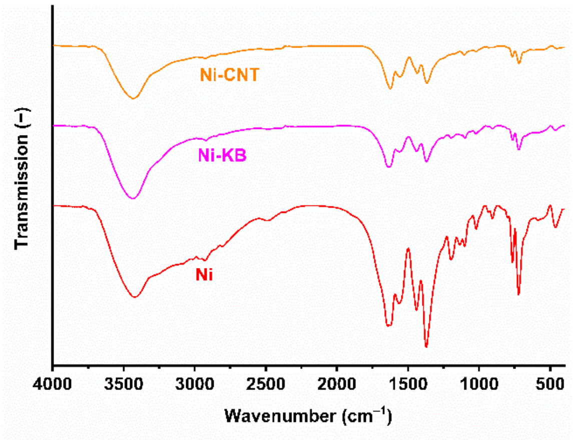

(a)

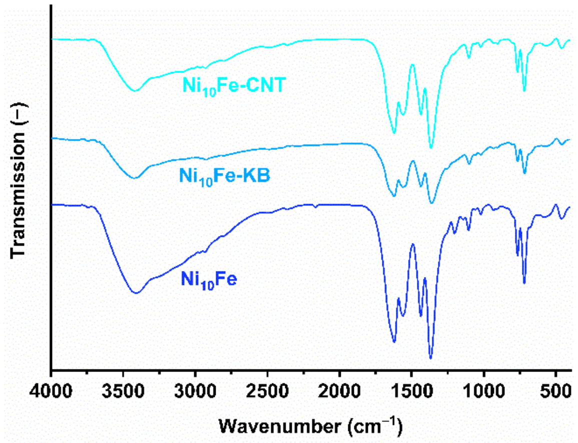

(b)

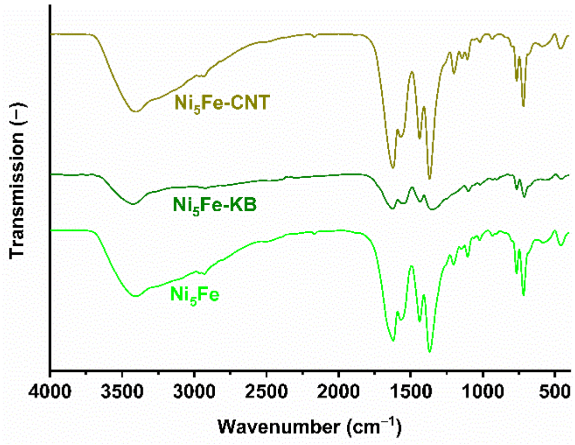

(c)

Figure S13. FT-IR spectra of neat MOFs and their in situ composites. **(a)** Ni-MOF, Ni-KB, Ni-CNT, **(b)** $Ni_{10}Fe$, $Ni_{10}Fe$-KB, $Ni_{10}Fe$-CNT, **(c)** Ni_5Fe, Ni_5Fe-KB and Ni_5Fe-CNT.

Table S3. Assignments of FT-IR-bands of neat MOFs and their composites (cm^{-1})

Assignment [1]	Ni-BTC	Ni-BTC-KB	Ni-BTC-CNT	$Ni_{10}Fe$-BTC	$Ni_{10}Fe$-BTC-KB	$Ni_{10}Fe$-BTC-CNT	Ni_5Fe-BTC	Ni_5Fe-BTC-KB	Ni_5Fe-BTC-CNT
**ν(OH) (of e.g. water)** [1,2,3]	3424	3437	3433	3411	3425	3420	3405	3428	3404
**ν(C-H)** [1–4]	2964	2924	2959	2970	—	2967	2963	—	2967
	2929	2855	2924	2933	2925	2930	2930	2924	2934
			2858						
**ν(N-H)** [2,3,5]	2805	2803	2806	—	2803	2803	—	2798	—
	2490	—	2480	2497	2489	2485	2509	2480	—
	2367	—	—	2363	—	2362	—	2346	—
	—	—	—	2167	—	—	2168	2294	2167
**ν_{as}(OCO)** [2–6,12]	1625	1629	1625	1621	1624	1622	1621	1625	1623
	1563	1562	1561	1562	1561	1563	1567	1559	1568
**ν_s(OCO)** [2–6,12]	1440	1439	1436	1437	1436	1437	1438	1434	1438
	1372	1371	1367	1369	1362	1366	1369	1345	1370
**ν(C-N) (of DMF)** [3,4] /	1198	1198	—	1203	—	—	1204	—	1201
	1136	—	—	1144	—	—	1148	—	1144
**ν(C-C)$_{Ar}$** [3,4]	1102	1101	1104	1106	1103	1104	1105	1101	1107
**ϱ(C-H)$_{Ar}$** [3,7]	—	—	—	1061	1063	1062	1062	—	1062
	1021	1022	1021	1021	1022	1021	1022	1019	1021
**ν(CN-CHO) (of DMF)** [3,8]	934	934	931	934	934	933	934	931	935
**δ(C-H)$_{Ar}$** [1,3,5,9]/	907	905	905	909	907	906	908	903	—
	799	—	—	—	802	—	—	803	798
**ν(C-C)$_{Ar}$** [3,5,9]	765	765	766	766	766	767	766	765	766
**ν(Fe_2Ni-O)** [3,10,11] / **γ (C-C)$_{Ar}$** [5]	722	721	719	720	717	719	718	713	719
**ν(Fe/Ni-O)** [3,9,12]	585	—	561	581	577	572	583	567	589
	463	465	453	462	461	461	459	456	463

[1] ν = stretching vibration (ν_{as}= asymmetric, ν_s = symmetric vibration); δ = bending vibration (ϱ = in plane, γ = out of plane vibration); Ar = aromatic vibration

Section S7. Porosity related parameters derived from N_2-soprtion measurements

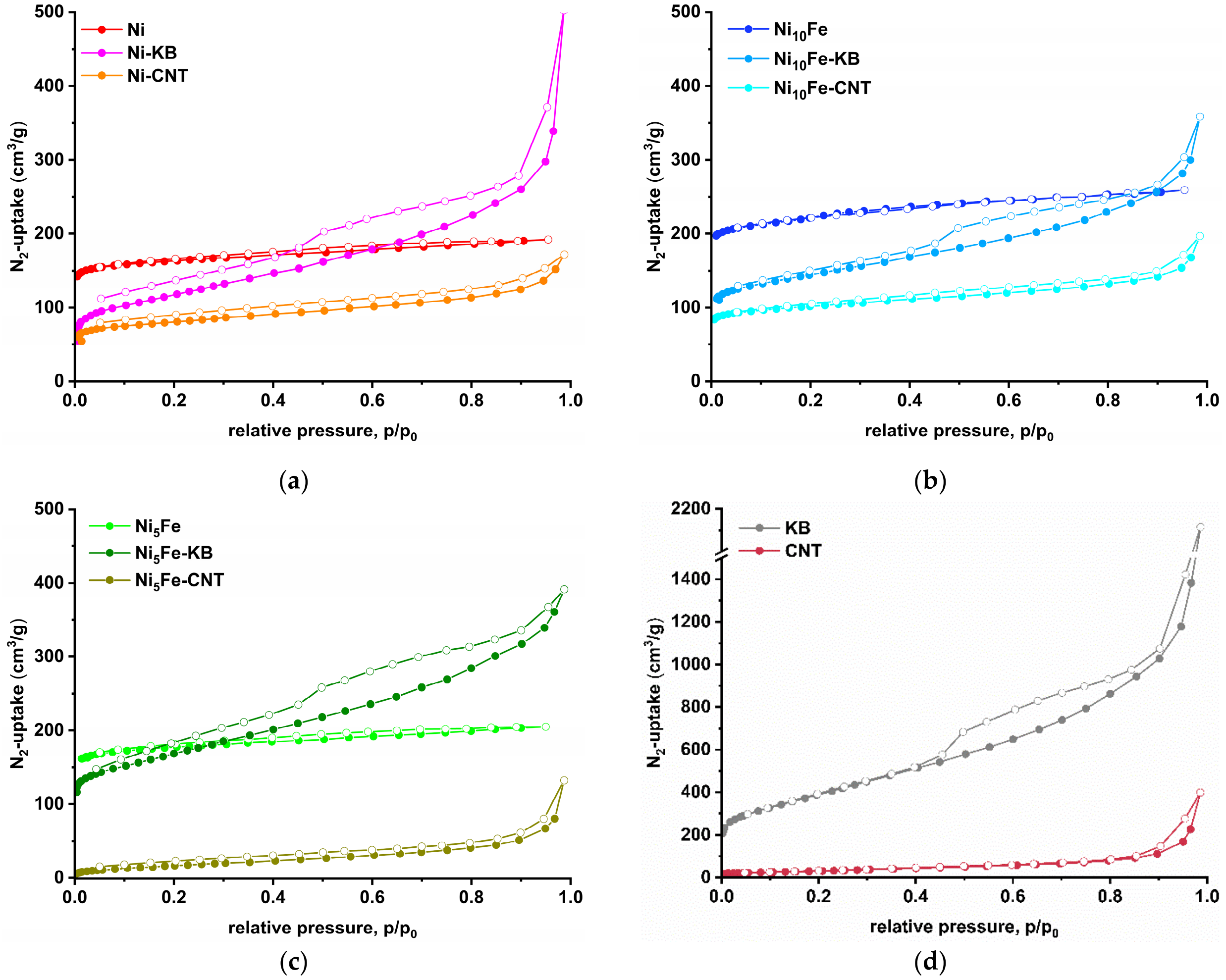

Figure S14. N_2-sorption isotherms neat MOFs and their in situ composites at 77 K (adsorption: filled circles; desorption: empty circles) **(a)** Ni-MOF, Ni-KB, Ni-CNT, **(b)** $Ni_{10}Fe$, $Ni_{10}Fe$-KB, $Ni_{10}Fe$-CNT, **(c)** Ni_5Fe, Ni_5Fe-KB, Ni_5Fe-CNT, **(d)** KB and CNT.

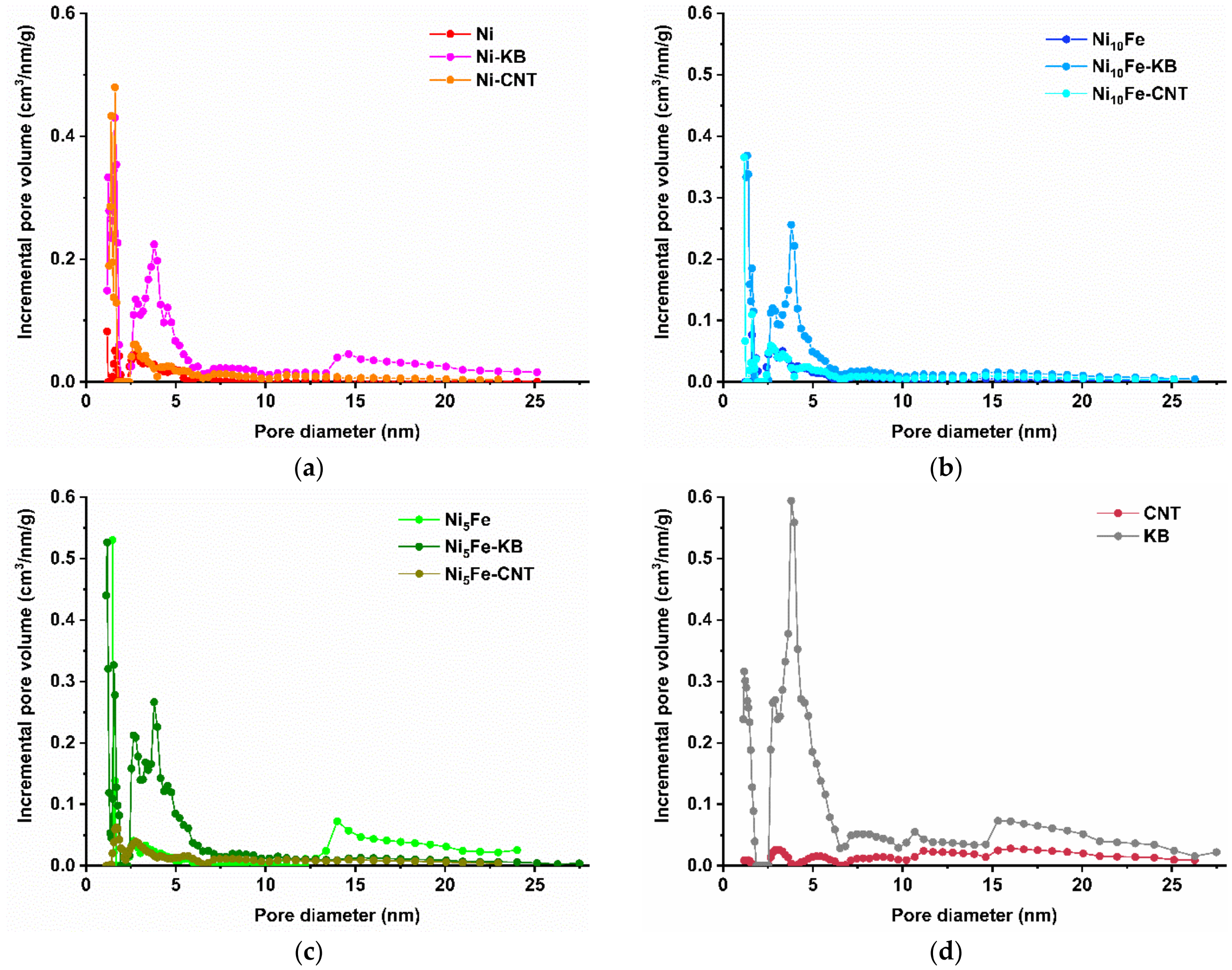

Figure S15. Pore size distributions of **(a)** Ni-MOF, Ni-KB, Ni-CNT, **(b)** Ni$_{10}$Fe, Ni$_{10}$Fe-KB, Ni$_{10}$Fe-CNT, **(c)** Ni$_5$Fe, Ni$_5$Fe-KB, Ni$_5$Fe-CNT, **(d)** KB and CNT.

Table S4. Porosity related parameters for all in situ Ni-MOF-carbon and Ni$_x$Fe-carbon samples derived from N_2-sorption measurements.

Sample (in situ syntheses)	BET surface area (m²/g) [1]	Total pore volume (cm³/g) [2]	Main pore width maximum (nm) [3]
			NLDFT
**Ni-MOF**	648	0.29	1.2
**Ni-KB**	414	0.46	1.6
**Ni-CNT**	301	0.21	1.6
**Ni$_{10}$Fe**	859	0.40	1.6
**Ni$_{10}$Fe-KB**	526	0.44	1.4
**Ni$_{10}$Fe-CNT**	387	0.24	1.6
**Ni$_5$Fe**	698	0.32	1.5
**Ni$_5$Fe-KB**	599	0.53	1.2
**Ni$_5$Fe-CNT**	67	0.10	1.7
**KB**	1415	1.59	3.8
**CNT**	117	0.17	—

[1] From N_2-sorption measurements at 77 K (Figure 5), calculated BET area based on the adsorption points between p/p_0 = 0.01–0.1. [2] Determined based on the adsorption at p/p_0 = 0.95. [3] Calculated by non-local density functional theory (NLDFT) with the model carbon with slit/cylindrical pores.

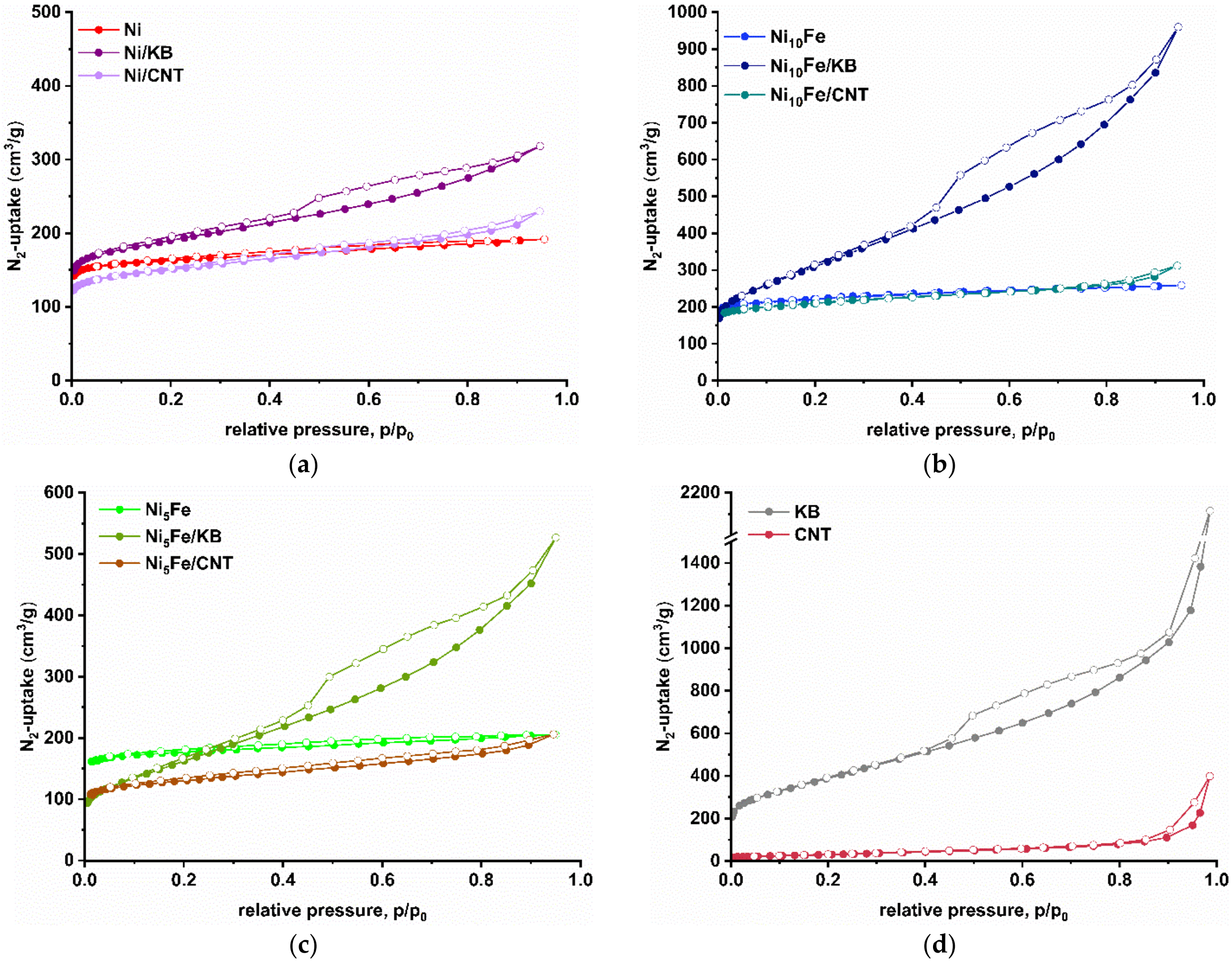

Figure S16. N_2-sorption isotherms neat MOFs and their postsynthetic composites at 77 K (adsorption: filled circles; desorption: empty circles) **(a)** Ni-MOF, Ni/KB, Ni/CNT, **(b)** $Ni_{10}Fe$, $Ni_{10}Fe$/KB, $Ni_{10}Fe$/CNT, **(c)** Ni_5Fe, Ni_5Fe/KB, Ni_5Fe/CNT, **(d)** KB and CNT.

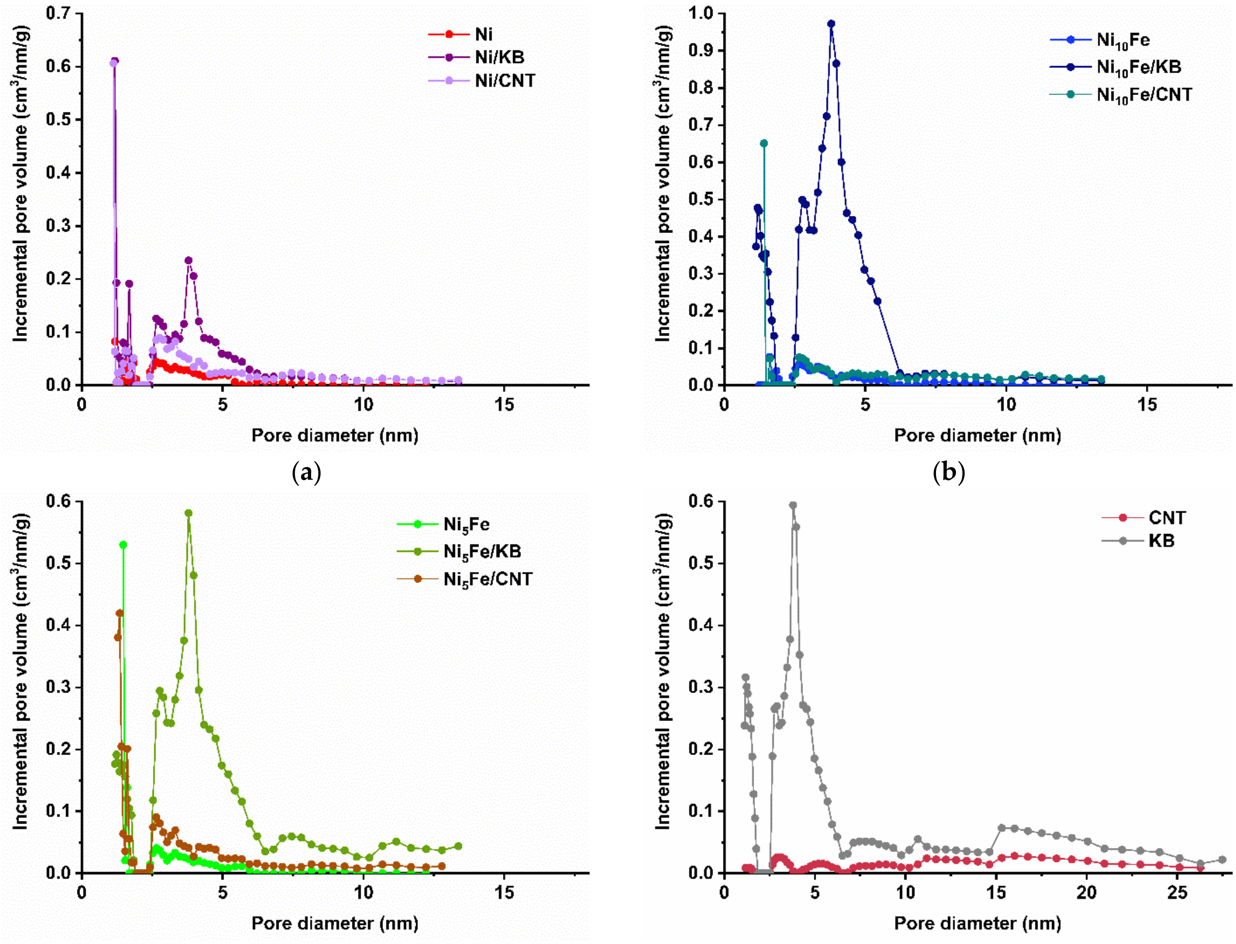

(c) (d)

Figure S17. Pore size distributions of **(a)** Ni, Ni/KB, Ni/CNT, **(b)** $Ni_{10}Fe$, $Ni_{10}Fe$/KB, $Ni_{10}Fe$/CNT, **(c)** Ni_5Fe, Ni_5Fe/KB, Ni_5Fe/CNT, **(d)** KB and CNT.

Table S5. Porosity related parameters for all postsynthetic Ni-MOF/carbon and Ni_xFe/carbon samples derived from N_2-sorption measurements.

Sample (postsynthetic mixtures)	BET surface area (m^2/g) [1]	Total pore volume (cm^3/g) [2]	Main pore width maximum (nm) [3]
			**NLDFT**
**Ni-MOF**	648	0.29	1.2
**Ni/KB**	712	0.47	1.2
**Ni/CNT**	570	0.33	1.1
**$Ni_{10}Fe$**	859	0.40	1.6
**$Ni_{10}Fe$/KB**	1060	1.29	1.2
**$Ni_{10}Fe$/CNT**	797	0.44	1.4
**Ni_5Fe**	698	0.32	1.5
**Ni_5Fe/KB**	604	0.69	1.2
**Ni_5Fe/CNT**	490	0.29	1.4
**KB**	1415	1.59	3.8
**CNT**	117	0.17	—

[1] From N_2-sorption measurements at 77 K (Figure S14), calculated BET area based on the adsorption points between p/p_0 = 0.01–0.1. [2] Determined based on the adsorption at p/p_0 = 0.95. [3] Calculated by non-local density functional theory (NLDFT) with the model carbon with slit/cylindrical pores.

Section S8. Electrochemical Data

Section S8.1. SEM and SEM-EDX for electrochemistry

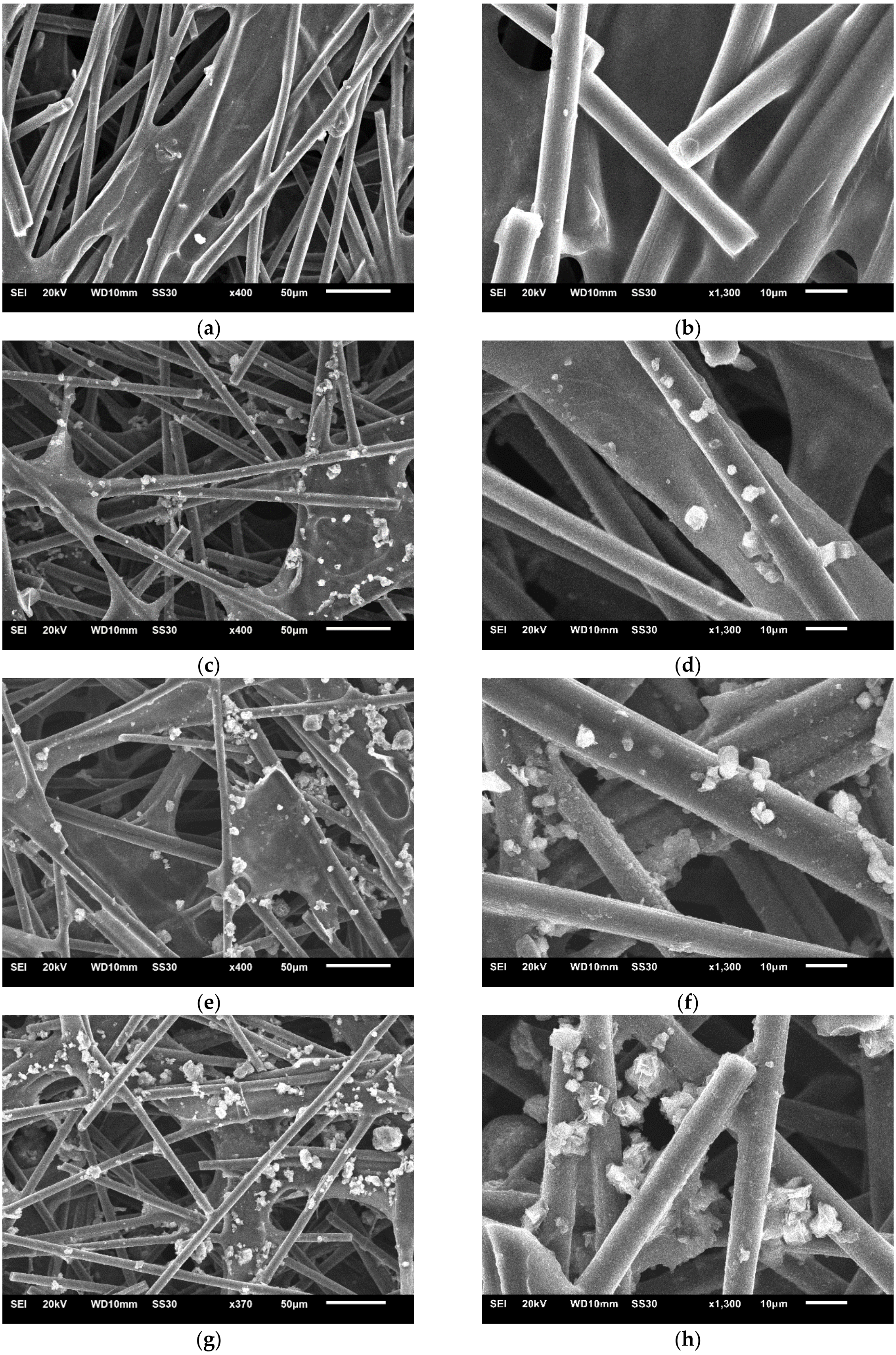

Figure S18. SEM images of carbon paper **(a-b)** without a sample containing ink, **(c-d)** with $Ni_{10}Fe$, **(e-f)** with $Ni_{10}Fe$-KB and **(g-h)** with $Ni_{10}Fe$-CNT containing ink before the electrochemical measurements.

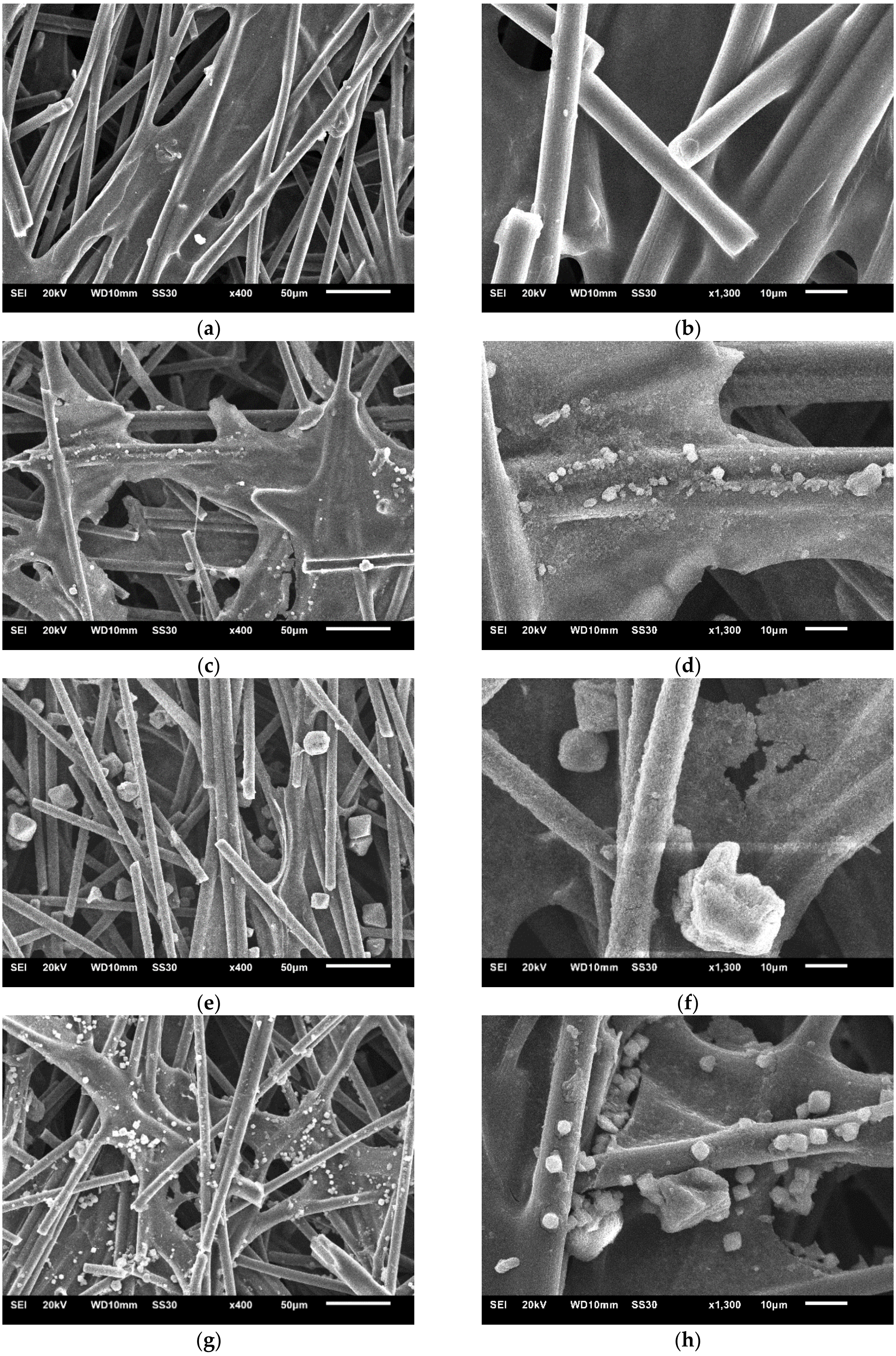

Figure S19. SEM images of carbon paper **(a-b)** without a sample containing ink, **(c-d)** with Ni_5Fe, **(e-f)** with Ni_5Fe-KB and **(g-h)** with Ni_5Fe-CNT containing ink before the electrochemical measurements.

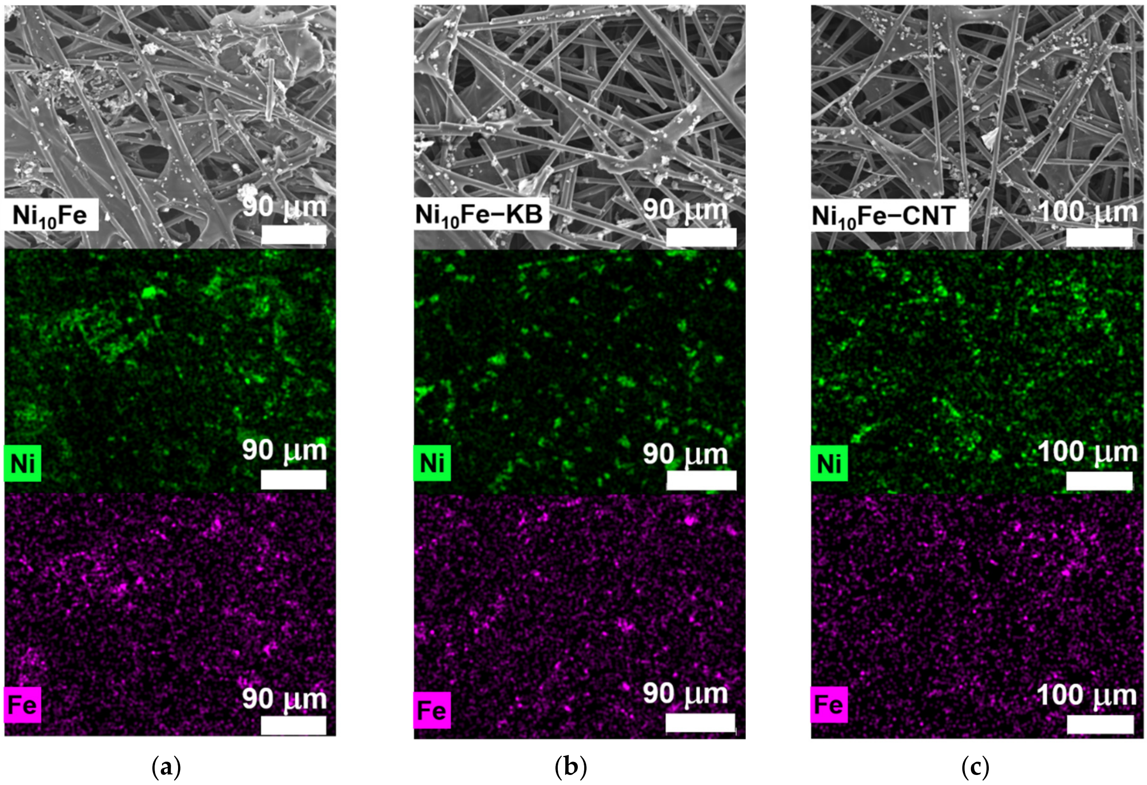

Figure S20. SEM images (first row) and EDX elemental mapping for Ni (second row) and Fe (third row) for **(a)** $Ni_{10}Fe$, **(b)** $Ni_{10}Fe$-KB and **(c)** $Ni_{10}Fe$-CNT on carbon paper before the electrochemical measurements.

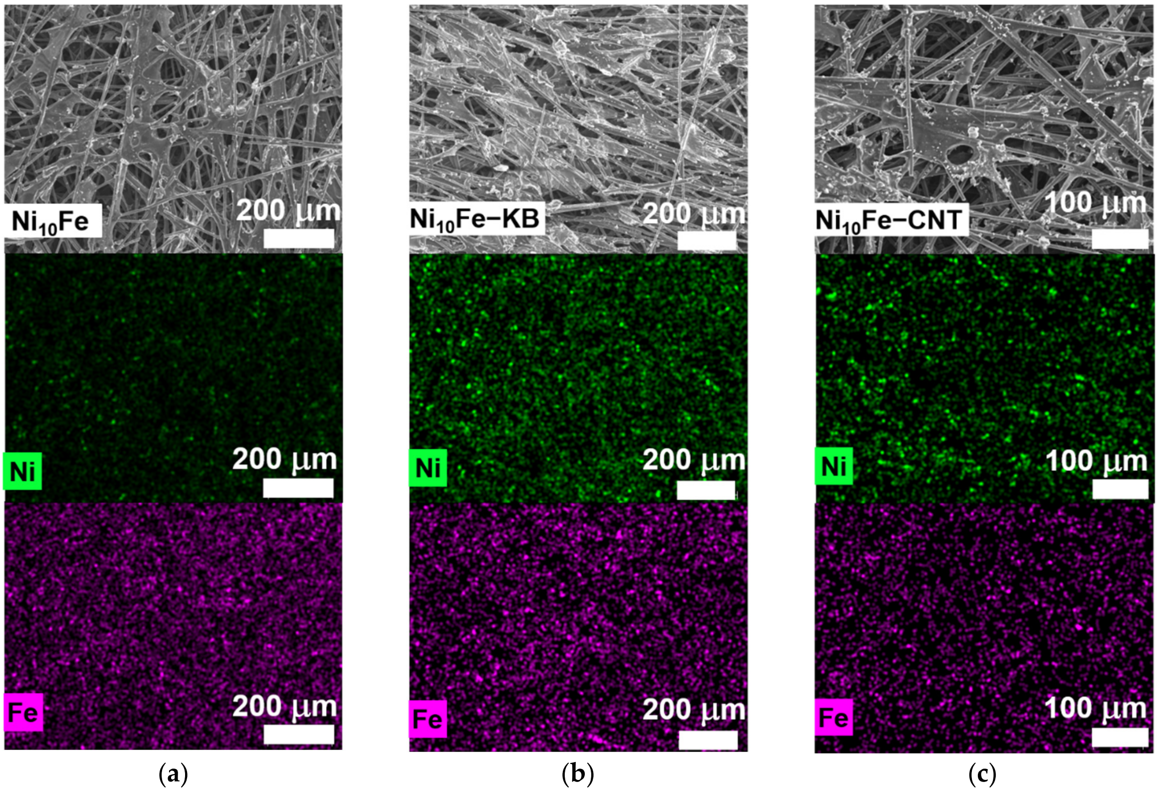

Figure S21. SEM images (first row) and EDX elemental mapping for Ni (second row) and Fe (third row) for **(a)** $Ni_{10}Fe$, **(b)** $Ni_{10}Fe$-KB and **(c)** $Ni_{10}Fe$-CNT on carbon paper after the electrochemical measurements.

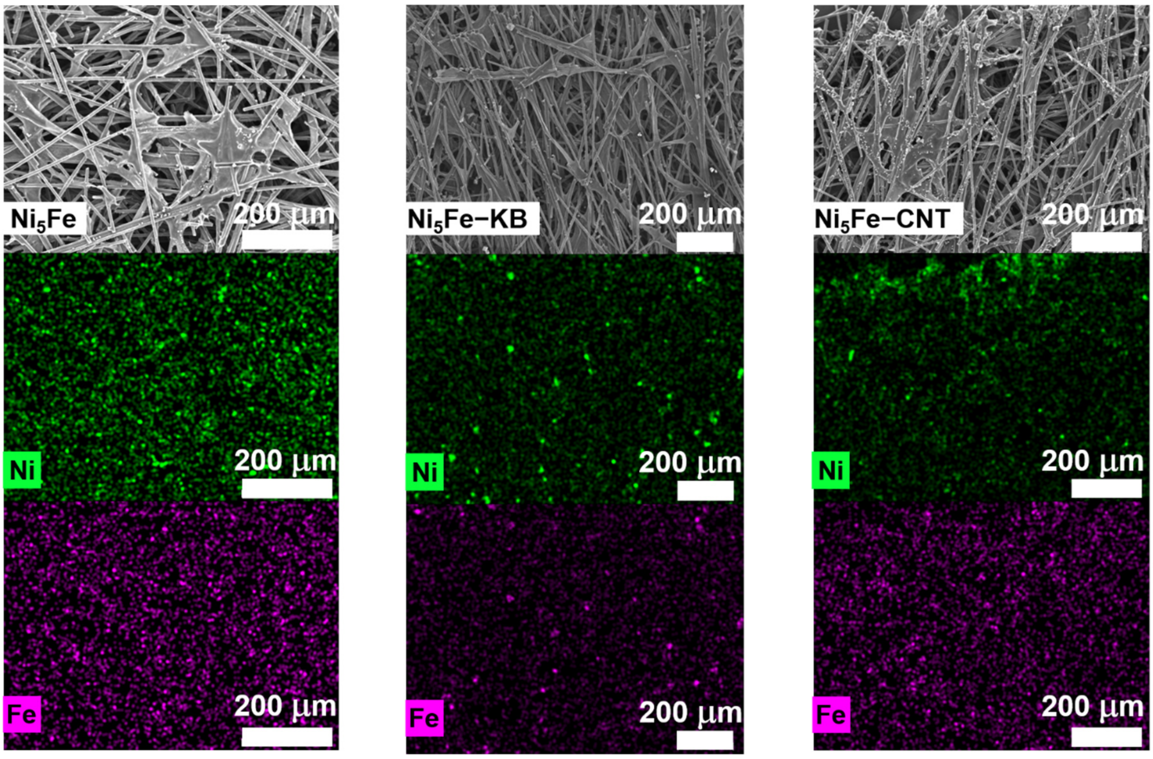

(**a**) (**b**) (**c**)

Figure S22. SEM images (first row) and EDX elemental mapping for Ni (second row) and Fe (third row) for **(a)** Ni_5Fe, **(b)** Ni_5Fe-KB and **(c)** Ni_5Fe-CNT on carbon paper before the electrochemical measurements.

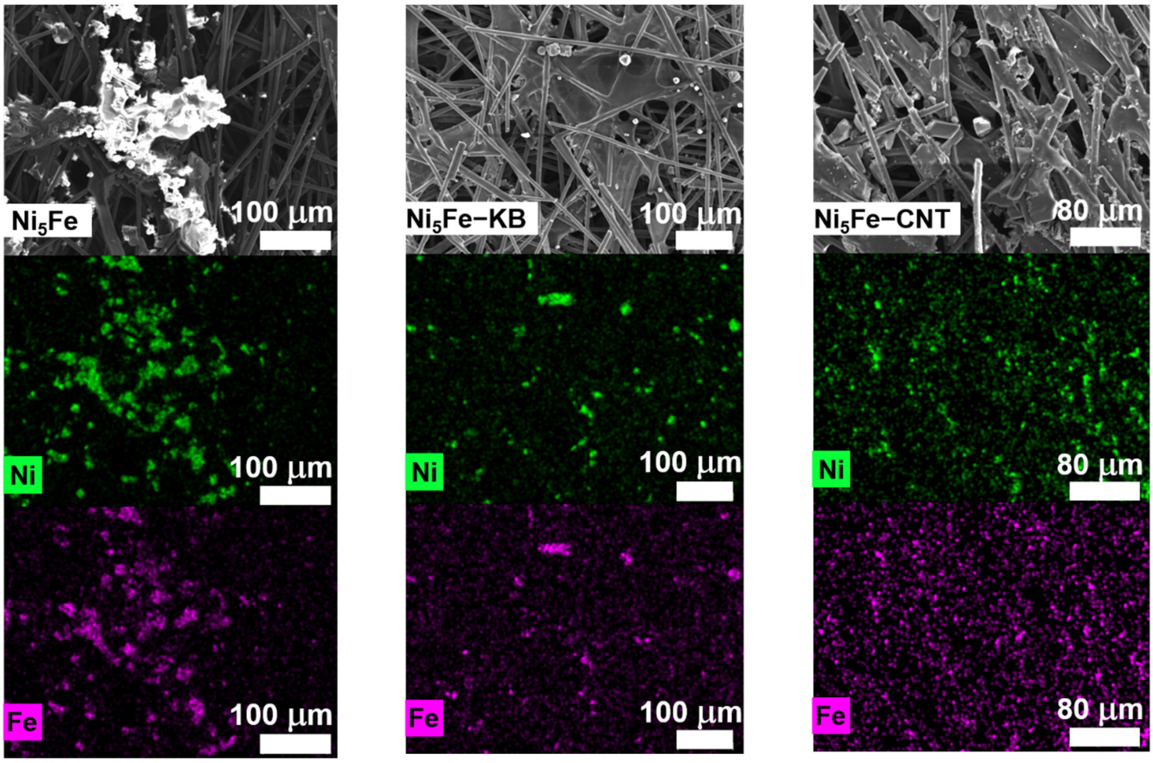

(**a**) (**b**) (**c**)

Figure S23. SEM images (first row) and EDX elemental mapping for Ni (second row) and Fe (third row) for **(a)** Ni_5Fe, **(b)** Ni_5Fe-KB and **(c)** Ni_5Fe-CNT on carbon paper after the electrochemical measurements.

Section S8.2. Overpotentials, Tafel slopes and charge transfer resistances

Table S6. Overpotentials at 10 mA/cm², Tafel slope and R_{CT}.

**Sample** [1]	**η @ 10 mA/cm² (mV) before → after 1000 cycles** [2]	**Tafel slope (mV/dec)**	**R_{CT} (Ω)**
**Ni-MOF**	372±3 → 379±5	100	201
**Ni-KB**	365±8 → 354±3	76	133
**Ni-CNT**	361±4 → 348±2	87	150
**$Ni_{10}Fe$**	311±5 → 320±7	72	20
**$Ni_{10}Fe$-KB**	322±7 → 339±7	70	33
**$Ni_{10}Fe$-CNT**	313±9 → 324±12	67	25
**Ni_5Fe**	296±3 → 314±6	70	10
**Ni_5Fe-KB**	314±6 → 333±2	64	15
**Ni_5Fe-CNT**	289±1 → 301±1	58	7
**Ni/KB**	351±2→ 341±4	76	82
**Ni/CNT**	354±2 → 351±4	75	90
**$Ni_{10}Fe$/KB**	313±2 → 314±5	68	17
**$Ni_{10}Fe$/CNT**	322±7 → 329±2	73	21
**Ni_5Fe/KB**	297±3 → 311±5	70	10
**Ni_5Fe/CNT**	294±5 → 312±5	68	10
**KB**	470 → N/A	134	235
**CNT**	450 → 441	137	1507
**RuO_2**	360±8 → 354±10	91	39

[1] The neat MOF samples are highlighted for clarity. [2] Error bars are derived from the highest and lowest achieved η_{10} value in the multiple measurements.

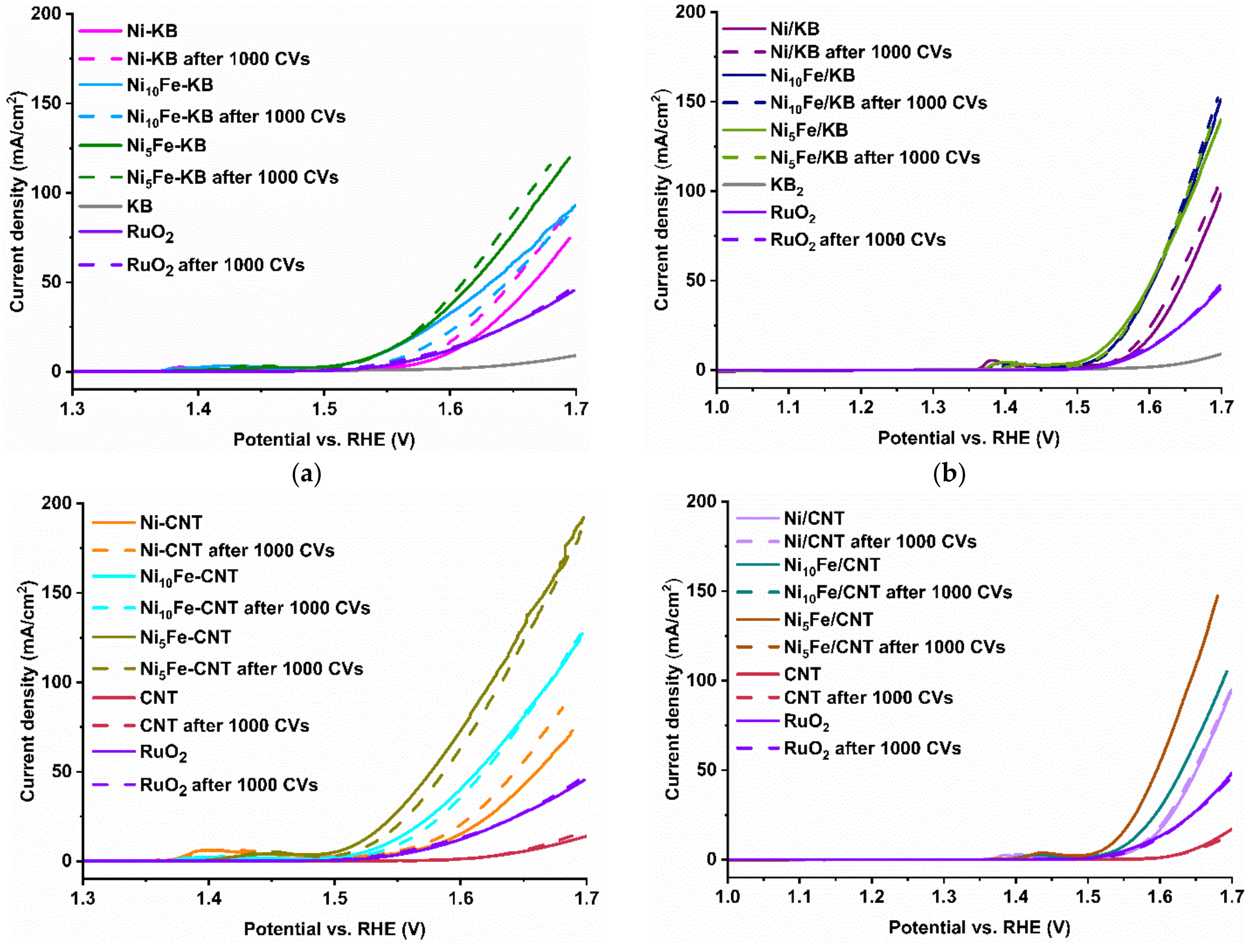

Figure S24. LSV plots before and after 1000 CVs of **(a)** the in situ and **(b)** the postsynthetic KB composites compared to KB and RuO_2 and **(c)** the in situ and **(d)** the postsynthetic CNT composites compared to CNT and RuO_2.

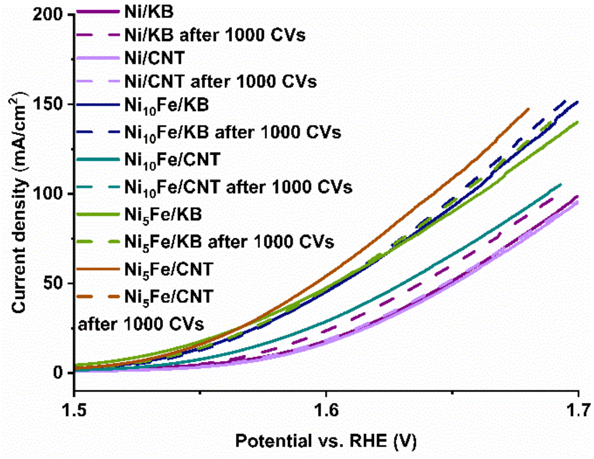

Figure S25. Enlargement of LSV plot before and after 1000 CVs of postsynthetic physical mixtures of Ni- and Ni_xFe-MOFs with KB or CNT.

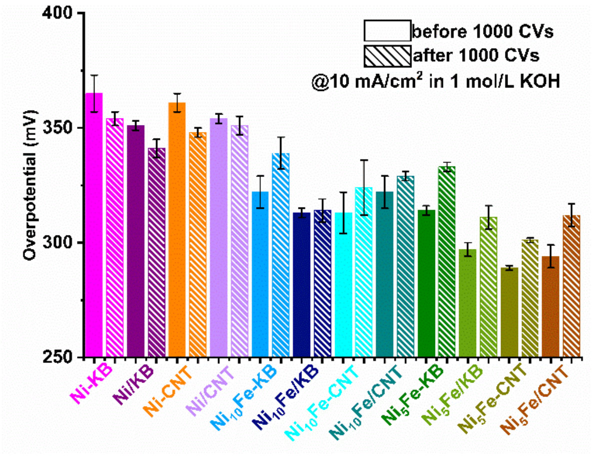

Figure S26. Overpotentials η_{10} calculated from LSV curves in Figure 3 (a-d) of in situ MOF-carbon samples and postsynthetic MOF/carbon mixtures for comparison. Error bars are derived from the highest and lowest achieved η_{10} value in the multiple measurements.

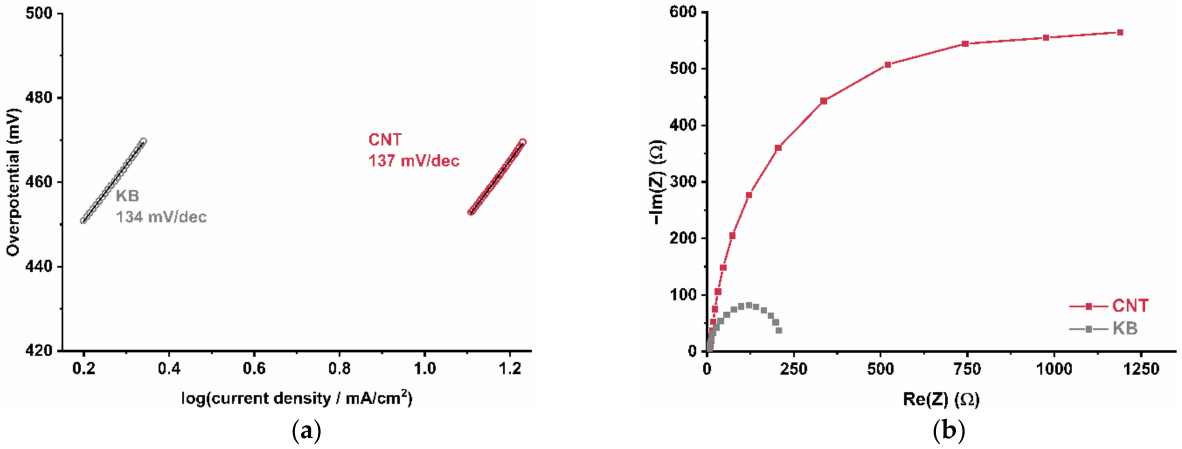

Figure S27. (a) Tafel plots and **(b)** Nyquist plots of KB and CNT.

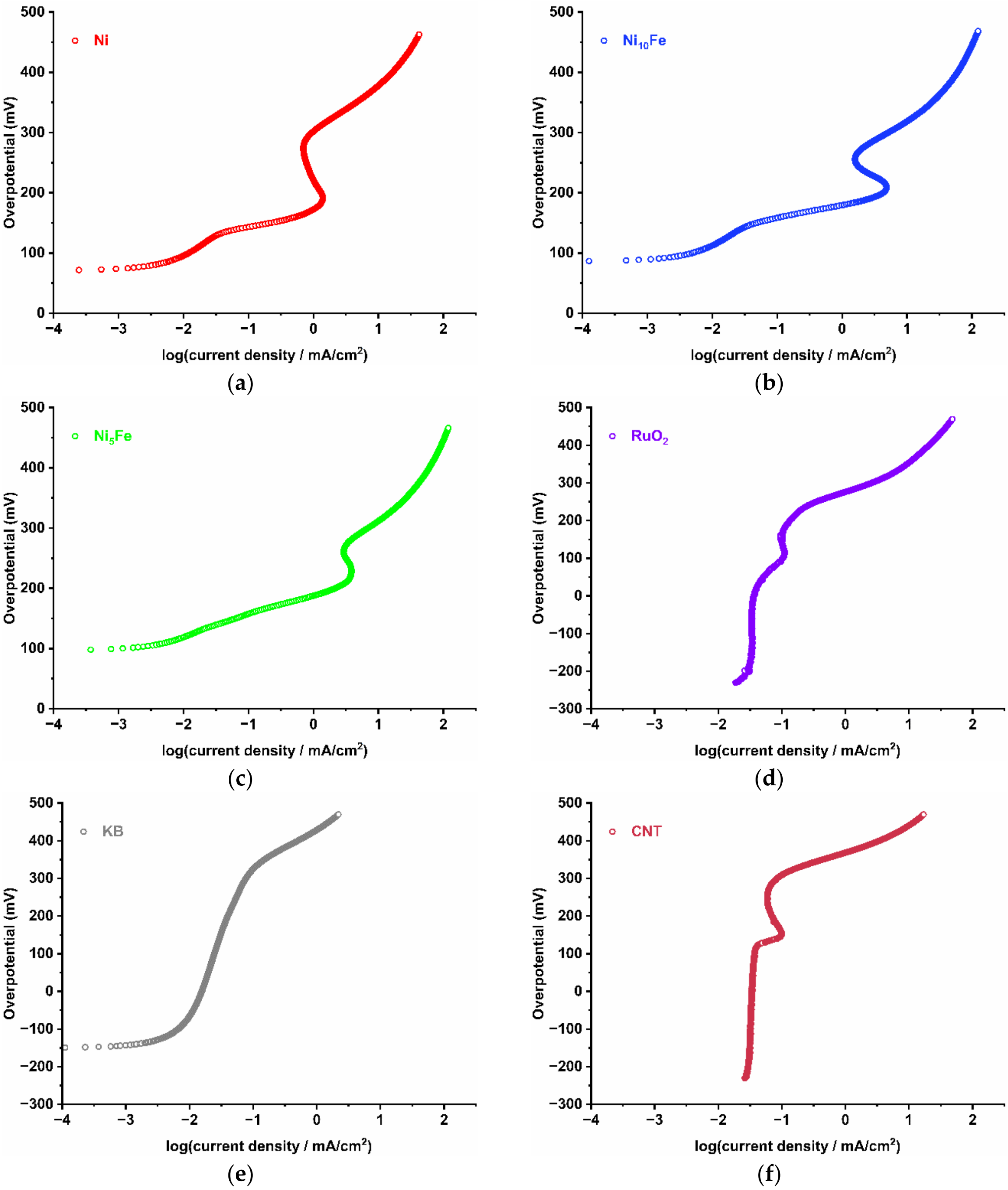

Figure S28. Tafel plots with the entire current-overpotential data of the MOFs, **(a)** Ni, **(b)** $Ni_{10}Fe$, **(c)** Ni_5Fe and of **(d)** RuO_2, **(e)** KB and **(f)** CNT. The plot for each sample is presented individually to be able to see the full dataset without any overlap from other samples.

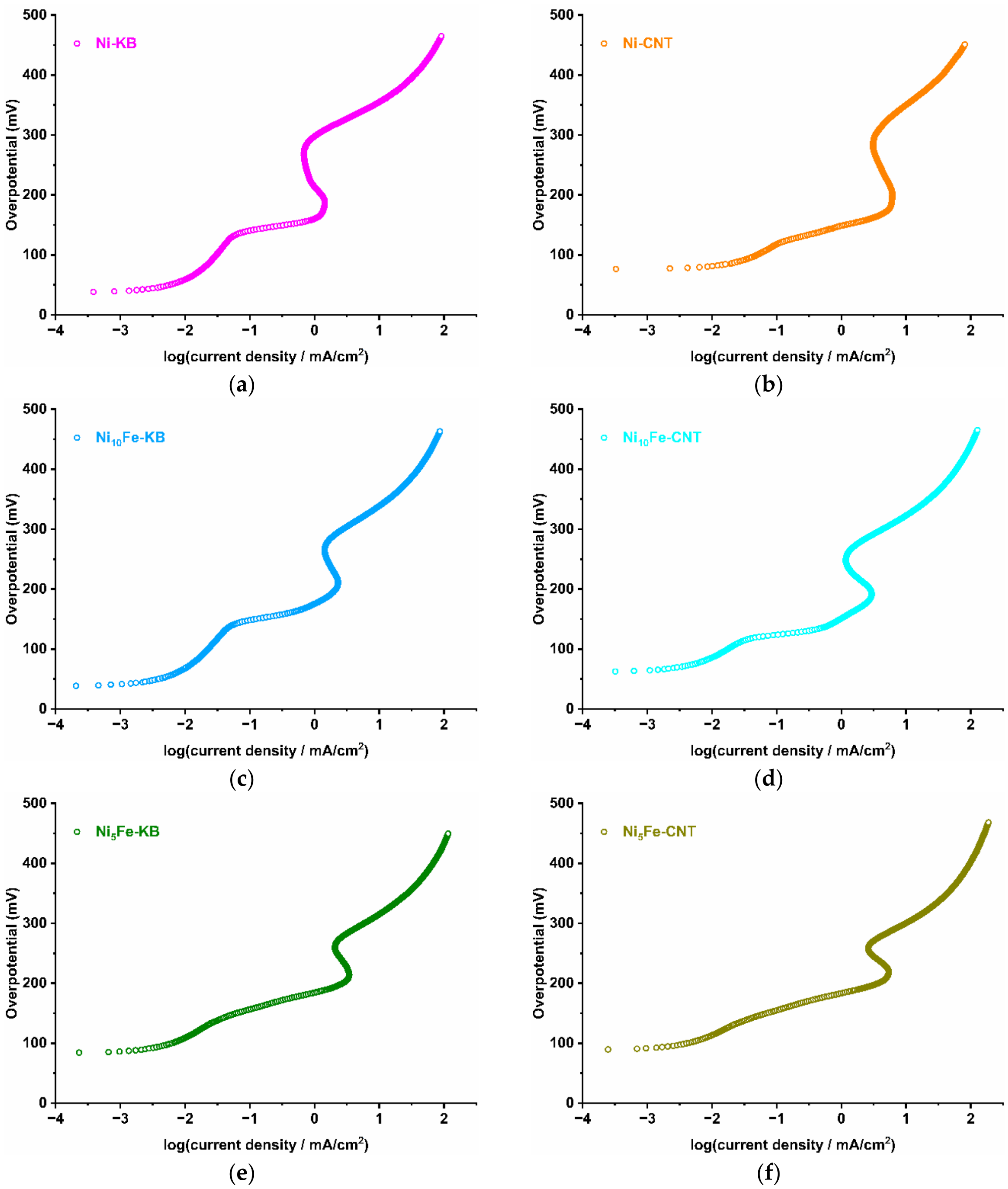

Figure S29. Tafel plots with the entire current-overpotential data of the in situ MOF-carbon composites: **(a)** Ni-KB, **(b)** Ni-CNT, **(c)** $Ni_{10}Fe$-KB, **(d)** $Ni_{10}Fe$-CNT, **(e)** Ni_5Fe-KB, **(f)** Ni_5Fe-CNT. The plot for each sample is presented individually to be able to see the full dataset without any overlap from other samples.

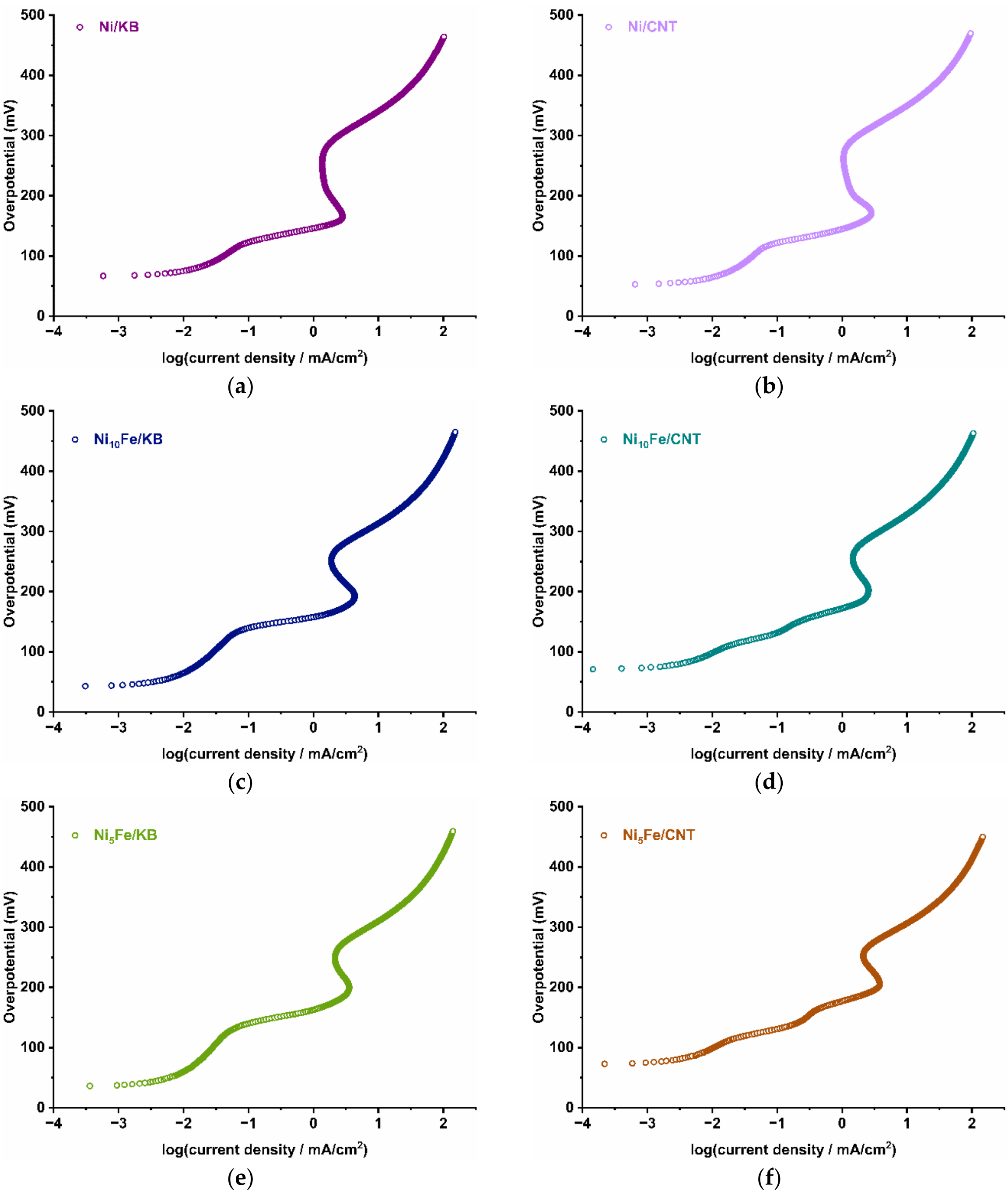

Figure S30. Tafel plots with the entire current-overpotential data of the postsynthetically mixed MOF/carbon composites: **(a)** Ni/KB, **(b)** Ni/CNT, **(c)** $Ni_{10}Fe$/KB, **(d)** $Ni_{10}Fe$/CNT, **(e)** Ni_5Fe/KB, **(f)** Ni_5Fe/CNT. The plot for each sample is presented individually to be able to see the full dataset without any overlap from other samples.

Section S8.3. Chronopotentiometry

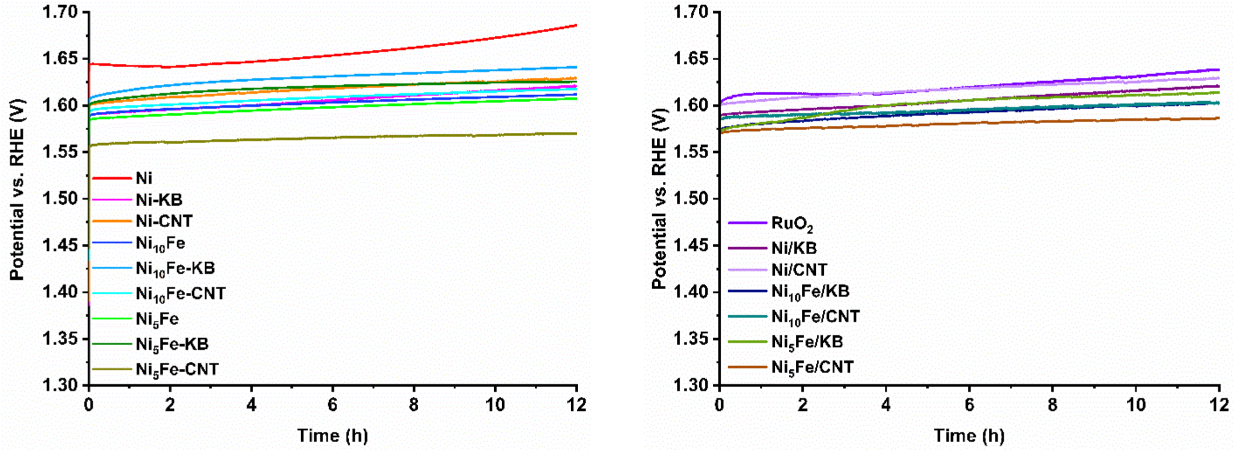

(**a**) (**b**)

Figure S31. Chronopotentiometry of **(a)** Ni-MOF, Ni-KB, Ni-CNT, $Ni_{10}Fe$, $Ni_{10}Fe$-KB, $Ni_{10}Fe$-CNT, Ni_5Fe, Ni_5Fe-KB, Ni_5Fe-CNT, **(b)** RuO_2 and postsynthetic mixtures of Ni-MOF, $Ni_{10}Fe$ and Ni_5Fe.

Section S8.4. Faradaic efficiencies (FE)

Table S7. Faradaic efficiencies.

Sample	FE (%) without correction	FE (%) with correction [1]
Ni_5Fe	> 100	96
Ni_5Fe-KB	> 100	94
Ni_5Fe/KB	> 100	92
Ni_5Fe-CNT	> 100	95
Ni_5Fe/CNT	> 100	86
KB	> 100	72
CNT	> 100	77
RuO_2	> 100	91
Bare carbon paper	60	0

[1] With correction by the detected percentage of oxygen from a measurement without any applied current to determine the oxygen portion coming from ambient air due to leaks in the system.

Section S8.5. Metal hydroxides

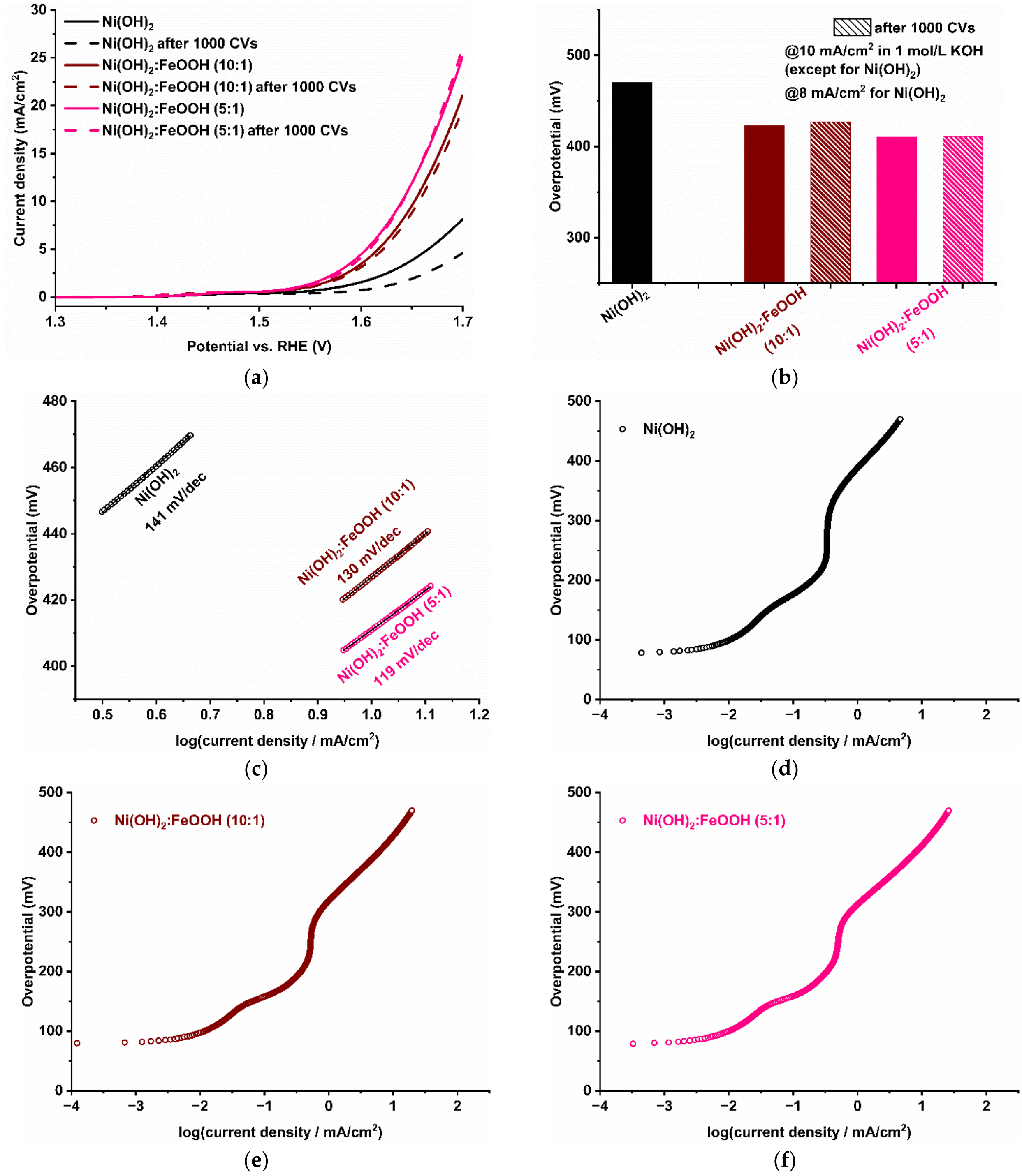

Figure S32. **(a)** LSV plot before and after 1000 CVs of $Ni(OH)_2$, $Ni(OH)_2$:FeOOH (10:1) and $Ni(OH)_2$:FeOOH (5:1) mixtures. **(b)** Overpotentials η_{10} (except for $Ni(OH)_2$ with η_8) calculated from LSV curves in Figure S32a. Tafel plots **(c)** with Tafel slope *b* (given in mV/dec) and **(d-f)** with the entire current-overpotential data of the metal hydroxides.

Table S8. Overpotentials at 10 mA/cm² and Tafel slopes of the metal hydroxides.

Sample	η @ 10 mA/cm² (mV)[1] before → after 1000 cycles	Tafel slope (mV/dec)
$Ni(OH)_2$	470 → N/A[1]	141
$Ni(OH)_2$:FeOOH 10:1	423 → 427	130
$Ni(OH)_2$:FeOOH 5:1	410 → 411	119

[1] Except for $Ni(OH)_2$ where η is given at 8 mA/cm²; N/A = not available

Section S8.6. Three-electrode setup

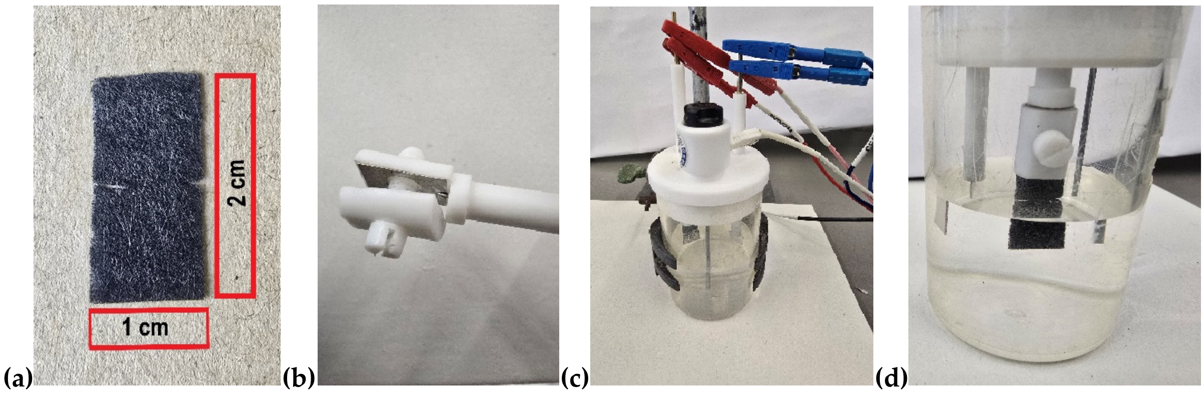

(a) (b) (c) (d)

Figure S33. (a) 2x1 cm carbon paper piece with a cut at 1 cm from both sides to define two 1x1 cm areas, **(b)** platinum-metal contact containing clamp, **(c)** three-electrode setup, **(d)** close-up picture of the electrochemical cell.

Section S9. References

1. Maniam, P.; Stock, N. Investigation of Porous Ni-Based Metal-Organic Frameworks Containing Paddle-Wheel Type Inorganic Building Units via High-Throughput Methods. *Inorg. Chem.* **2011**, *50*, 5085-5097. DOI: 10.1021/ic200381f
2. Meier, H.; Bienz, S.; Bigler, L.; Fox, T. Spektroskopische Methoden in der organischen Chemie, 9th ed., Georg Thieme, Stuttgart, Germany, **2016**.
3. Sondermann, L.; Jiang, W.; Shviro, M.; Spieß, A.; Woschko, D.; Rademacher, L.; Janiak, C. Nickel-based metal-organic frameworks as electrocatalysts for the oxygen evolution reaction (OER). *Molecules* **2022**, *27*, 1241. DOI: 10.3390/molecules27041241
4. Wade, C. R.; Dincă, M. Investigation of the synthesis, activation, and isosteric heats of CO_2 adsorption of the isostructural series of metal–organic frameworks $M_3(BTC)_2$ (M = Cr, Fe, Ni, Cu, Mo, Ru). *Dalton Trans.* **2012**, *41*, 7931-7938. DOI: 10.1039/c2dt30372h
5. Wu, Y.; Song, X.; Li, S.; Zhang, J.; Yang, X.; Shen, P.; Gao, L.; Wei, R.; Zhang, J.; Xiao, G. 3D-monoclinic M–BTC MOF (M = Mn, Co, Ni) as highly efficient catalysts for chemical fixation of CO_2 into cyclic carbonates. *J. Ind. Eng. Chem.* **2018**, *58*, 296-303. DOI: 10.1016/j.jiec.2017.09.040
6. Zhang, M.; Hu, D.; Xu, Z.; Liu, B.; Boubeche, M.; Chen, Z.; Wang, Y.; Luo, H.; Yan, K. Facile synthesis of Ni-, Co-, Cu-metal organic frameworks electrocatalyst boosting for hydrogen evolution reaction. *J. Mater. Sci. Technol.* **2021**, *72*, 172–179. DOI: 10.1016/j.jmst.2020.09.028
7. Pretsch, E.; Bühlmann, P.; Badertscher, M. Spektroskopische Daten Zur Strukturaufklärung organischer Verbindungen, 5th ed.; Springer: Berlin/Heidelberg, Germany, **2010**.
8. Israr, F.; Chun, D.; Kim, Y.; Kim, D. K. High yield synthesis of Ni-BTC metal–organic framework with ultrasonic irradiation: Role of polar aprotic DMF solvent. *Ultrason. Sonochem.* **2016**, *31*, 93–101. DOI:10.1016/j.ultsonch.2015.12.007
9. Chau, N. T. K.; Chung, Y.-M. Ethylene oligomerization over mesoporous FeNi-BTC catalysts: Effect of the textural properties of the catalyst on the reaction performance *Mol. Catal.* **2023**, *541*, 113094. DOI: 10.1016/j.mcat.2023.113094
10. Vuong, G.-T.; Pham, M.-H.; Do, T.-O. Synthesis and engineering porosity of a mixed metal Fe_2Ni MIL-88B metal–organic framework. *Dalton Trans.* **2013**, *42*, 550–557. DOI: 10.1039/c2dt32073h
11. Yaqoob, L.; Noor, T.; Iqbal, N.; Nasir, H.; Zaman, N.; Talha, K. Electrochemical synergies of Fe–Ni bimetallic MOF CNTs catalyst for OER in water splitting. *J. Alloys Compd.* **2021**, *850*, 156583. DOI: 10.1016/j.jallcom.2020.156583
12. Arul, P.; Abraham John, S. Size controlled synthesis of Ni-MOF using polyvinylpyrrolidone: New electrode material for the trace level determination of nitrobenzene. *J. Electroanal. Chem.* **2018**, *829*, 168–176. DOI: 10.1016/j.jelechem.2018.10.014
